# Chronic physical conditions and risk for perinatal mental illness: A population-based retrospective cohort study

**DOI:** 10.1371/journal.pmed.1002864

**Published:** 2019-08-26

**Authors:** Hilary K. Brown, Andrew S. Wilton, Joel G. Ray, Cindy-Lee Dennis, Astrid Guttmann, Simone N. Vigod

**Affiliations:** 1 Interdisciplinary Centre for Health & Society, University of Toronto Scarborough, Toronto, Canada; 2 Dalla Lana School of Public Health, University of Toronto, Toronto, Canada; 3 Department of Psychiatry, University of Toronto, Toronto, Canada; 4 ICES, Toronto, Canada; 5 Women’s College Research Institute, Women’s College Hospital, Toronto, Canada; 6 Li Ka Shing Knowledge Institute, St. Michael’s Hospital, Toronto, Canada; 7 Lawrence S. Bloomberg Faculty of Nursing, University of Toronto, Toronto, Canada; 8 Hospital for Sick Children, Toronto, Canada; 9 Department of Paediatrics, University of Toronto, Toronto, Canada; King's College London, UNITED KINGDOM

## Abstract

**Background:**

One in 5 women experience mental illness in pregnancy or post partum. Universal preventive interventions have not lowered the incidence of perinatal mental illness, perhaps because those at highest risk were not targeted. Outside of pregnancy, chronic physical conditions are known to confer increased risk for mental illness. Our objective was to examine the association between chronic physical conditions and risk of perinatal mental illness.

**Methods and findings:**

We conducted a population-based retrospective cohort study using linked health administrative data sets in Ontario, Canada, in 2005 to 2015. We compared 77,385 women with chronic physical conditions to 780,619 women without such conditions, all of whom had a singleton live birth. Excluded were women with a mental illness diagnosis within 2 years before pregnancy. Chronic physical conditions were captured using the Agency for Healthcare Research and Quality Chronic Condition Indicator, applied to acute healthcare encounters in the 2 years before pregnancy. The outcome was perinatal mental illness, defined by a mental illness or addiction diagnosis arising between conception and 365 days post partum. The outcome was further defined by timing (prenatal or post partum) and specific diagnosis (psychotic disorder, mood or anxiety disorder, substance use disorder, self-harm, or other). Modified Poisson regression generated relative risks and 95% confidence intervals (CIs), adjusted for age, parity, rural residence, income quintile, and remote history of mental health care. Women in the cohort had an average age of 29.6 years (standard deviation 5.4), 44.2% were primiparous, 11.0% lived in a rural area, 40.1% were in the lowest 2 income quintiles, and 47.9% had a remote history of mental health care. More women with (20.4%) than without (15.6%) a chronic physical condition experienced perinatal mental illness—an adjusted relative risk (aRR) of 1.20 (95% CI 1.18–1.22, *p* < 0.0001). The aRRs were statistically significant for mental illness in pregnancy (1.12, 95% CI 1.10–1.15, *p* < 0.0001) and post partum (1.25, 95% CI 1.23–1.28, *p* < 0.0001). Psychotic disorders (aRR 1.50, 95% CI 1.36–1.65, *p* < 0.0001), mood or anxiety disorders (aRR 1.19, 95% CI 1.17–1.21, *p* < 0.0001), substance use disorders (aRR 1.47, 95% CI 1.34–1.62, *p* < 0.0001), and other mental illness (aRR 1.68, 95% CI 1.50–1.87, *p* < 0.0001) were more likely in women with than without chronic physical conditions, but not self-harm (aRR 1.14, 95% CI 0.87–1.48, *p* = 0.34). The study was limited by reliance on acute health care encounters to measure chronic physical conditions and the inability to capture undiagnosed mental health problems.

**Conclusions:**

Findings from this study suggest that women with a chronic physical condition predating pregnancy may be at heightened risk of developing mental illness in the perinatal period. These women may require targeted efforts to lower the severity of their condition and improve their coping strategies and supports in pregnancy and thereafter.

## Abstract 2

Hilary Brown and colleagues reveal that a chronic physical illness that predates pregnancy increases the risk of perinatal depression.

## Author summary

**Why was this study done?:**

- Despite prevention efforts, 1 in 5 women experience mental illness in pregnancy or post partum.
- Outside of pregnancy, chronic physical conditions such as diabetes, hypertension, and asthma are known to increase the risk of mental illness.
- The association between chronic physical conditions and mental illness in the perinatal period has rarely been studied.

**What did the researchers do and find?:**

- We used health administrative data for 77,385 women with chronic physical conditions and 780,619 women without such conditions in Ontario, Canada, to compare their risks of a psychotic disorder, mood or anxiety disorder, substance use disorder, self-harm, or another mental illness arising in pregnancy or within 365 days of childbirth.
- We found that more women with (20.4%) than without (15.6%) a chronic physical condition experienced perinatal mental illness.
- This risk was largely consistent across the timing and specific diagnosis of perinatal mental illness as well as the type of chronic physical condition.

**What do these findings mean?:**

- Women with chronic physical conditions may require specific efforts to impact disease management and improve their coping strategies during pregnancy and post partum.
- These types of interventions could be implemented before pregnancy to prevent perinatal mental illness.

## Introduction

One in 5 women suffer from a mental illness during pregnancy or within the year thereafter [1]. Perinatal mental illness negatively affects mothers, infants, and families [2,3], but only one-third of women with perinatal mental illness receive mental health care [4]. Morbidity and societal cost could be averted by early identification and treatment [5]. Although stress and low social support are among the strongest predictors of perinatal mental illness [6], universal preventive interventions developed to address these factors have not substantially reduced rates of perinatal mental illness [5]. A better understanding of those at higher risk of perinatal mental illness would inform targeted prevention strategies.

The prevalence of chronic physical conditions, including diabetes, hypertension, and asthma, in pregnancy has risen over time, in parallel with higher maternal age and obesity: nearly 20% of pregnant women in high-income countries have a chronic physical condition [7]. Research in nonpregnant populations suggests that individuals with chronic physical conditions are at higher risk for mental illness than those without such conditions [8–11]. This is partly explained by psychosocial factors, such as stress arising from disease management, and biological factors, such as inflammation [12,13]. The perinatal period is a major life transition, and psychosocial stressors related to disease management among women with chronic physical conditions may be exacerbated in this period. They may also experience problems related to pre-existing disease or obstetrical complications. Therefore, one might posit that chronic physical conditions might increase a woman’s vulnerability to perinatal mental illness.

The existing literature on the association between chronic physical conditions and perinatal mental illness is limited. A recent meta-analysis of 12 studies showed that chronic physical conditions were associated with an increased odds of 1.43 for perinatal mental illness [14]. However, the studies included therein were limited by the fact that none examined the development of a psychotic disorder, substance use disorder, or self-harm. Furthermore, because few studies excluded women with pre-existing mental illness, they could not delineate whether chronic physical conditions were associated with incident or ongoing perinatal mental illness. Some studies in the meta-analysis may also have unnecessarily adjusted for variables that lie along the causal pathway between chronic physical conditions and perinatal mental illness, including foetal and newborn complications [15,16].

The objective of the current study was to examine the association between maternal chronic physical conditions predating conception and the risk of perinatal mental illness diagnosed in pregnancy or up to 1 year thereafter.

## Methods

### Study design and setting

This was a population-based retrospective cohort study in Ontario, Canada. With 14 million residents and over 140,000 obstetrical deliveries every year, Ontario is Canada’s largest province [17]. Essential health care services, including all primary and acute care, obstetrical care, and mental health care with a physician, are delivered at no direct cost to residents.

The current study cohort considered women aged 15 to 49 years with a live birth conceived between April 1, 2005 and March 31, 2015. Women were followed for 365 days post partum to ascertain study outcomes, to a maximum date of December 31, 2016. Excluded were women with diagnosed mental illness in the 2-year period before conception in the index pregnancy. A mental illness diagnosis was based on one or more physician or emergency department visits or a hospitalization for any psychotic disorder, mood or anxiety disorder, substance use disorder, self-harm event, or any other mental illness.

This study is reported as per the Strengthening the Reporting of Observational Studies in Epidemiology (STROBE) guideline (S1 Checklist). A prospective analysis plan was used to design the study (S1 Text). The use of data was authorized under section 45 of Ontario’s Personal Health Information Protection Act, which does not require review by a research ethics board.

### Data sources

We accessed and analysed health administrative data at ICES, in Toronto, Ontario. ICES captures sociodemographic and diagnostic data, from the healthcare encounters of Ontario residents, that are linked at the individual level using a unique, encoded identifier. Women with a singleton livebirth were identified in the MOMBABY data set, which links >98% of maternal and newborn records for the delivery hospitalization [17]. The date of conception was determined by subtracting the recorded gestational age—generally based on dating from a first trimester ultrasound [18]—from the recorded date of birth. We also used the Canadian Institute for Health Information Discharge Abstract Database for hospitalizations, the Ontario Mental Health Reporting System for psychiatric hospitalizations, the National Ambulatory Care Reporting System for emergency department visits, the Ontario Health Insurance Plan (OHIP) Database for outpatient visits, the Client Agency Program Enrolment Database for a registry of patients enrolled in primary care groups, the ICES Physician Database for physician specialties, and the Registered Persons Database for sociodemographic characteristics. Information in the OHIP Database is recorded using physician billing claims; information in hospital databases is recorded using the Canadian Coding Standards for the International Statistical Classification of Diseases and Related Health Problems, 10th revision (ICD-10) or Diagnostic and Statistical Manual of Mental Disorders, 4th edition (DSM-IV). The information in these databases has been shown to be complete and reliable; sociodemographic data, physician billing claims, and primary diagnoses in hospital data sets have excellent completeness and accuracy [19].

### Exposure

Women in the exposed group were those with one or more chronic physical conditions. Our conceptualization of chronic physical conditions was based on the Agency for Healthcare Research and Quality Chronic Condition Indicator for the ICD-10 [20]. Conditions were defined as chronic according to whether they last 12 months or longer and (1) limit independent living, social interactions, or self-care or (2) require ongoing intervention with medical services, products, or equipment [20]. Chronic physical conditions were grouped by body system, according to ICD-10 chapters: endocrine, nutritional, and metabolic diseases and immunity disorders; diseases of the circulatory system, respiratory system, musculoskeletal system, nervous system and sense organs, digestive system, genitourinary system, skin and subcutaneous tissue, and blood and blood-forming organs; neoplasms; infections and parasitic disease; congenital anomalies; and injuries (S1 Table).

We considered a woman to have a chronic physical condition if any ICD-10 code included in this definition was present in an acute healthcare encounter in the 24 months prior to conception, denoted either as an emergency department visit without a hospitalization or a hospitalization. Women in the unexposed group were those with no acute healthcare encounter for such a condition during the 24-month period preceding conception. Although women with mild or well-controlled conditions may be more likely to present to a primary care provider than an acute care setting, this conceptualization captures those with the most severe conditions, which would be expected to carry the greatest risk for perinatal mental illness.

### Outcomes

Perinatal mental illness was defined as at least 1 mental illness diagnosis between conception and 365 days post partum, captured by one of the following encounters: (1) a visit to a general practitioner or family physician with a mental illness diagnosis, (2) a visit to a psychiatrist, (3) an emergency department visit with a mental illness diagnosis, or (4) a hospitalization with a mental illness diagnosis [21,22]. Perinatal mental illness was further categorized by (a) its timing (i.e., prenatally, with the first encounter occurring during pregnancy; or post partum, with the first encounter occurring within 365 days of the delivery date) and (b) the diagnosis, namely, a psychotic disorder, mood or anxiety disorder, substance use disorder, another mental illness, or self-harm. Classification of the mental illness diagnosis was determined based on all healthcare encounters in the follow-up period, because women could have more than one perinatal mental illness diagnosis during this time (S2 Table) [21,22]. Because women with a recent history of mental illness in the 2 years before conception were excluded, it was reasonable to assume that perinatal mental illness arising in the index pregnancy largely represented a new episode of mental illness.

### Covariates

Covariates were social and health characteristics hypothesized to distinguish women with and without chronic physical conditions and that were available within the administrative data sets: maternal age, parity, neighbourhood income quintile, rural residence, and remote history of mental healthcare. Neighbourhood income quintile and rural residence are derived by linking residential postal code with census data [23]. Rural residences are those in communities of <10,000 individuals. A remote history of mental healthcare was defined as having 1 or more physician visits, emergency department visits, or hospitalizations for a psychotic disorder, mood or anxiety disorder, substance use disorder, self-harm, or another mental illness more than 2 years before the date of conception in the index pregnancy.

### Data analyses

Baseline characteristics of women with and without chronic physical conditions were described using means and proportions. Differences were expressed using standardized differences, with a value more than 0.10 determined to be clinically meaningful [24].

Unadjusted relative risks (RRs) and adjusted RRs (aRRs), and 95% confidence intervals (CIs), contrasted the risk of perinatal mental illness between women with versus without a prepregnancy chronic physical condition, using modified Poisson regression [25]. Because women may have delivered more than once during the study period, we accounted for potential clustering of births within mothers using generalized estimating equations [26]. All multivariable models were adjusted for maternal age, parity, rural residence, neighbourhood income quintile, and remote history of mental healthcare.

We conducted several prespecified additional analyses. One additional analysis examined the relationship between chronic physical conditions and perinatal mental illness by (a) the timing of its diagnosis (i.e., prenatally or post partum, as described above) and (b) the specific diagnosis of mental illness (as described above and in S2 Table). A second additional analysis explored the relationship between chronic physical conditions and perinatal mental illness by the number and type of body systems affected by chronic physical conditions (S1 Table). We also explored the relationship between different types of body systems affected by chronic physical conditions and (a) timing and (b) specific diagnosis of perinatal mental illness. For these analyses, also included in the multivariable models was the presence of other chronic physical conditions. Third, a more rigorous definition of the study outcome was instituted, requiring at least 2 physician visits for a mental health reason within pregnancy or 365 days post partum.

All data analyses used SAS version 9.4 (SAS Institute, Cary, North Carolina).

## Results

During the study period, there were 1,155,494 singleton livebirths. Of these, 297,490 (25.7%) were among women with a recent history of mental healthcare and were, therefore, excluded. The final study cohort comprised 858,004 singleton livebirths to 630,883 unique women. Nearly 1 in 10 women (9.0%) had a chronic physical condition (S3 Table). Women with a chronic physical condition versus no chronic physical condition tended to be slightly younger (28.9 versus 29.7 years), to live in a rural area (16.4% versus 10.5%), and to have a remote history of mental health care (56.4% versus 47.0%) (Table 1).

**Table 1:** Baseline characteristics of women with and without a chronic physical condition in the 24 months prior to conception. All data are presented as a number (%) unless otherwise specified.

Characteristic*	Chronic physical condition	No chronic physical condition	Standardized difference
	(*N* = 77,385)	(*N* = 780,619)	
**Age, years**			
**Mean (SD)**	28.9 (5.8)	29.7 (5.3)	0.14
**15–19**	4,763 (6.2)	29,861 (3.8)	0.11
**20–24**	13,905 (18.0)	99,496 (12.7)	0.15
**25–29**	22,195 (28.7)	238,682 (30.6)	0.04
**30–34**	22,751 (29.4)	271,708 (34.8)	0.12
**35–39**	11,430 (14.8)	120,052 (15.4)	0.02
**40–44**	2,224 (2.9)	19,968 (2.6)	0.02
**45–49**	117 (0.2)	852 (0.1)	0.01
**Primiparous**	36,772 (47.5)	342,280 (43.8)	0.07
**Rural residence**	12,698 (16.4)	81,594 (10.5)	0.18
**Neighbourhood income quintile**			
**Q1 (lowest)**	18,479 (23.9)	160,128 (20.5)	0.08
**Q2**	16,019 (20.7)	155,850 (20.0)	0.02
**Q3**	15,455 (20.0)	160,942 (20.6)	0.02
**Q4**	15,393 (19.9)	168,050 (21.5)	0.04
**Q5 (highest)**	11,565 (14.9)	131,552 (16.9)	0.05
**Remote history of mental health care more than 2 years preceding the index birth**	43,642 (56.4)	367,128 (47.0)	0.19

*Because women could have more than one livebirth during the study period, the level of analysis is the birth. Women could have different characteristics for different births.

**Abbreviation:** Q, quintile

Out of 77,385 women with a pre-existing chronic physical condition, 15,764 (20.4%) were diagnosed with a perinatal mental illness between conception and 1 year post partum, in contrast with 121,764 out of 780,619 women without a pre-existing chronic physical condition (15.6%)—equivalent to an unadjusted RR of 1.29 (95% CI 1.27–1.31, *p* < 0.0001; Table 2). The risk remained statistically significant after adjusting for confounders (aRR 1.20, 95% CI 1.18–1.22, *p* < 0.0001).

**Table 2:** Risk of perinatal mental illness arising between conception and 1 year post partum, in relation to a woman having a chronic physical condition in the 24 months prior to conception.

Variable	Number (%) with outcome	Unadjusted		Adjusted^a^	
		RR (95% CI)	*p*-value	RR (95% CI)	*p*-value
**Main exposure of interest**					
No chronic physical condition (*N* = 780,619)	121,764 (15.6)	1.00 (referent)		1.00 (referent)	
Chronic physical condition (*N* = 77,385)	15,764 (20.4)	1.29 (1.27–1.31)	<0.0001	1.20 (1.18–1.22)	<0.0001
**Age, years**					
15–24	30,348 (20.5)	1.34 (1.33–1.36)	<0.0001	1.35 (1.34–1.37)	<0.0001
25–34	83,965 (15.1)	1.00 (referent)		1.00 (referent)	
35–49	23,215 (15.0)	0.99 (0.97–1.00)	0.03	0.97 (0.96–0.98)	<0.0001
**Parity**					
Primiparous	66,634 (17.6)	1.00 (referent)		1.00 (referent)	
Multiparous	70,892 (14.8)	0.87 (0.86–0.88)	<0.0001	0.87 (0.86–0.88)	<0.0001
**Region of residence**					
Urban	123,485 (16.2)	1.00 (referent)		1.00 (referent)	
Rural	14,024 (14.9)	0.93 (0.91–0.95)	<0.0001	0.89 (0.87–0.90)	<0.0001
**Neighbourhood income quintile**					
Q1 (lowest)	30,610 (17.1)	1.12 (1.11–1.14)	<0.0001	1.09 (1.07–1.11)	<0.0001
Q2	28,049 (16.3)	1.07 (1.06–1.09)	<0.0001	1.05 (1.04–1.07)	<0.0001
Q3	28,075 (15.9)	1.05 (1.03–1.07)	<0.0001	1.04 (1.02–1.05)	<0.0001
Q4	28,499 (15.5)	1.03 (1.01–1.04)	0.002	1.02 (1.01–1.04)	0.005
Q5 (highest)	21,603 (15.1)	1.00 (referent)		1.00 (referent)	
**Remote history of mental health care more than 2 years preceding the index birth**					
Absent	52,868 (11.8)	1.00 (referent)		1.00 (referent)	
Present	84,660 (20.6)	1.70 (1.69–1.72)	<0.0001	1.75 (1.74–1.77)	<0.0001

^a^Adjusted for age, parity, rural residence, neighbourhood income quintile, and remote history of mental healthcare.

**Abbreviation:** CI, confidence interval; Q, quintile; RR, relative risk

**Fig 1:**
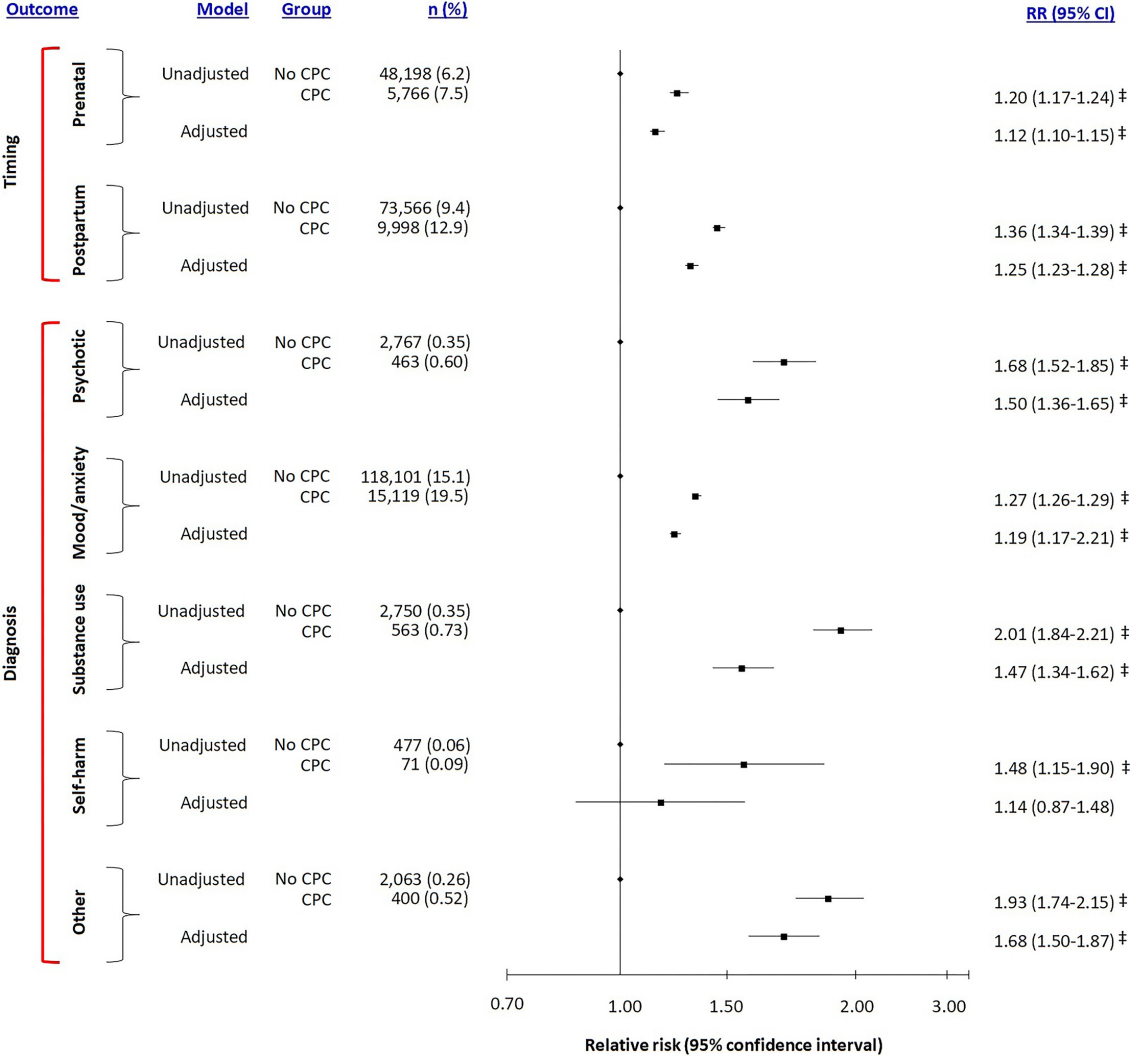
Risk of perinatal mental illness arising between conception and 1 year post partum, in relation to a woman having a chronic physical condition in the 24 months prior to conception, and further detailed by the timing and diagnosis of the perinatal mental illness. RRs were adjusted for maternal age, parity, rural residence, neighbourhood income quintile, and remote history of mental healthcare. *p < 0.05, †*p* < 0.01, ‡*p* < 0.001. CI, confidence interval; CPC, chronic physical condition; RR, relative risk.

In the first additional analysis, the absolute risk and aRR were higher for mental illness arising in the postpartum period (aRR 1.25, 95% CI 1.23–1.28, *p* < 0.0001) than for that diagnosed in pregnancy (aRR 1.12, 95% CI 1.10–1.15, *p* < 0.0001; Fig 1, Timing). The aRR was notably elevated for psychotic disorders (aRR 1.50, 95% CI 1.36–1.65, *p* < 0.0001), mood or anxiety disorders (aRR 1.19, 95% CI 1.17–1.21, *p* < 0.0001), substance use disorders (aRR 1.47, 95% CI 1.34–1.62, *p* < 0.0001), and other mental illness (aRR 1.68, 95% CI 1.50–1.87, *p* < 0.0001), but not for self-harm (Fig 1, Diagnosis).

**Fig 2:**
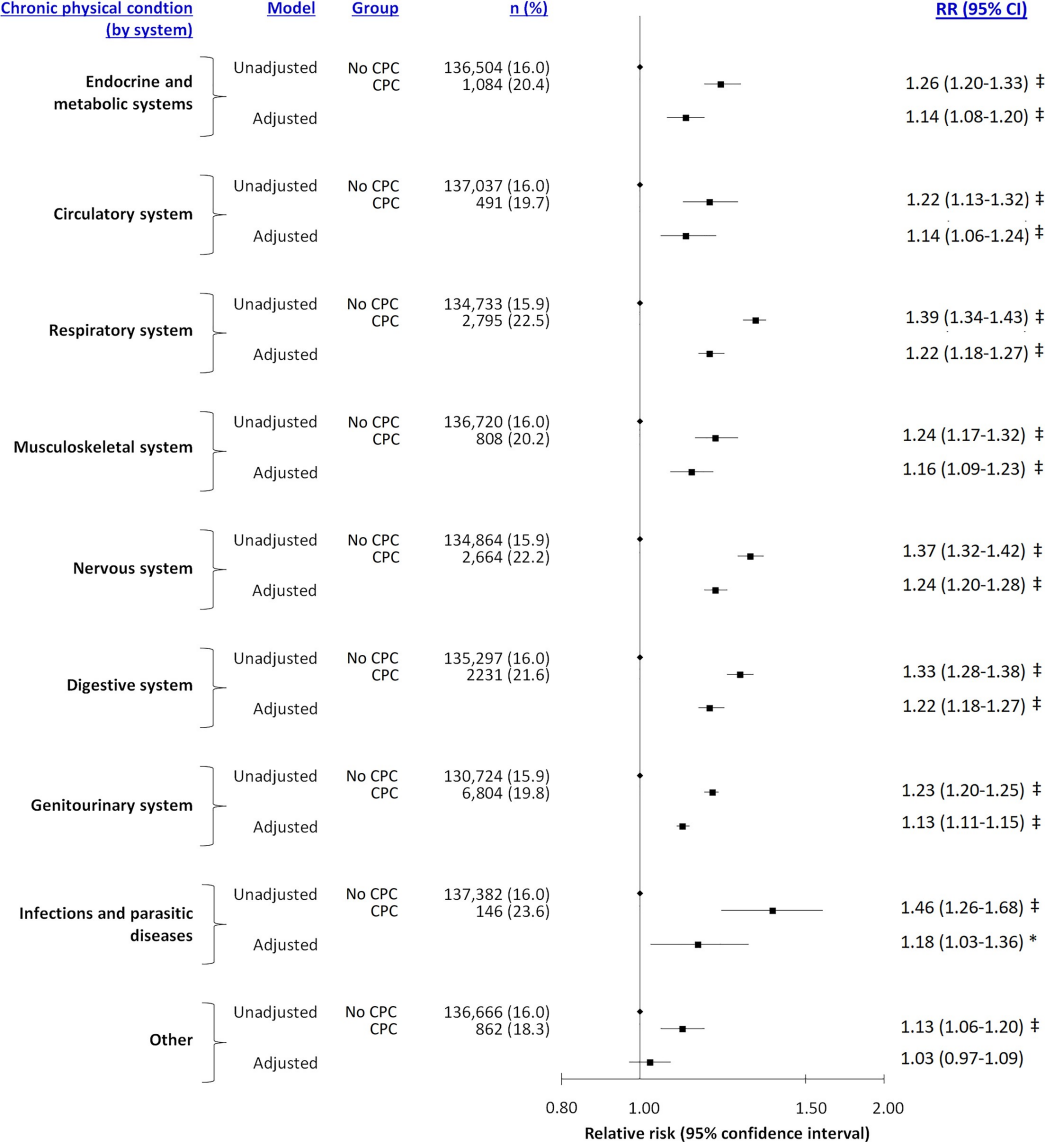
Risk of perinatal mental illness arising between conception and 1 year post partum, in relation to a woman having a chronic physical condition in the 24 months prior to conception, and further detailed by the type of chronic physical condition. RRs were adjusted for maternal age, parity, rural residence, neighbourhood income quintile, remote history of mental healthcare, and the presence of other chronic physical conditions. *p < 0.05, †*p* < 0.01, ‡*p* < 0.001. CI, confidence interval; CPC, chronic physical condition; RR, relative risk.

In the second additional analysis, women who had chronic physical conditions affecting more than 1 body system had higher absolute rates of perinatal mental illness (23.0%) than those with a solitary chronic physical condition (20.1%). Relative to women without a chronic physical condition, the corresponding aRRs were 1.30 (95% CI 1.25–1.35, *p* < 0.0001) and 1.19 (95% CI 1.17–1.21, *p* < 0.0001). The aRRs were also elevated across chronic physical conditions affecting different body systems, except for “other body systems” (Fig 2 and S4 Table). Findings were largely consistent by timing (S5 Table) and specific diagnosis of perinatal mental illness (S6 and S7 Tables).

In the third additional analysis, using a more rigorous definition of the study outcome of at least 2 physician visits for a mental health reason within pregnancy or 365 days post partum, women with a chronic physical condition (9.7%) were more likely than those without a chronic physical condition (6.5%) to have the outcome, equivalent to an unadjusted RR of 1.47 (95% CI 1.43–1.50, *p* < 0.0001) and an aRR of 1.31 (95% CI 1.28–1.34, *p* < 0.0001; S8 Table).

## Discussion

In this study of Canadian population-based health administrative data from a cohort of women with a singleton live birth and no recent history of mental illness in the 2 years before pregnancy, our results show that women with chronic physical conditions before pregnancy had a higher associated risk of new-onset perinatal mental illness, whether arising in pregnancy or post partum. This risk remained higher for all specific mental illness diagnoses except self-harm and was largely consistent across conditions affecting different body systems.

These findings are consistent with prior studies completed among nonpregnant groups, in which individuals with chronic physical conditions had a higher risk of mental illness [8–11]. Among those done in pregnancy, only a few studies distinguished between new-onset and pre-existing mental illness [27–31]. However, the current study also adjusted for remotely diagnosed mental illness, while excluding women with a recent diagnosis within 2 years preceding the index pregnancy’s date of conception. Like the findings of a previous meta-analysis, women with chronic physical conditions were observed to be at increased risk for mental illness arising within or after pregnancy, including depression and anxiety [14]. The current study further showed that women with chronic physical conditions were at increased risk for psychotic disorders and substance use disorders. Although the majority of prior studies focused on a single chronic physical condition, most commonly diabetes [32–34], the current study observed a higher risk of perinatal mental illness across conditions affecting most body systems and in the presence of chronic physical conditions affecting more than one body system.

There are several possible explanations for the association observed herein. In nonpregnant populations, stress related to chronic physical condition management, sleeplessness, or pain was thought to heighten the progression to mental illness [12]. These 3 factors may be exacerbated in the perinatal period, when women also experience body changes, hormonal factors, adjustment to motherhood, and infant care. In nonpregnant adults, diabetes and cardiovascular disease are associated with obesity, which may increase mental illness risk [35]. There is also evidence of shared genetic factors between diabetes and mental illness [36]. Autoimmune diseases, such as multiple sclerosis, may flare post partum, also increasing the risk of perinatal mental illness [37]. More generally, many chronic physical conditions are associated with hypothalamic-pituitary-adrenal axis dysfunction, elevated pro-inflammatory cytokines, and vascular pathology, all of which are implicated in the pathophysiology of mental illness [12]. Finally, there is evidence that the association between chronic physical conditions and perinatal mental illness is bi-directional, with individuals with mental illness having elevated rates of diabetes, cardiovascular disease, and other chronic conditions [38]. We tried to handle such reverse causation by excluding women with a recent history of mental illness and adjusting for a remote history of mental illness in the models. Nevertheless, mental illness can remit and relapse [6]. It is unclear why self-harm was the only outcome not significantly associated with chronic physical conditions. Possible explanations include the small number of events or the inability to capture self-harm behaviours not presenting in a healthcare context.

This study suggests that targeted management strategies might be needed among women with a chronic physical condition as a means to lower their risk of perinatal mental illness. Women with a chronic physical condition are generally already embedded within the healthcare system around the time of pregnancy, so there exist efficient avenues for care interventions partnering primary and obstetric care providers, along with a social worker or other mental health expert [39,40]. Interventions might be initiated preconceptionally, to support healthy behaviours and reduce modifiable risk factors, including obesity [41]. Screening, and other secondary prevention strategies in pregnancy and the postpartum period, may also be warranted. Our findings, along with the plausible psychosocial and biological mechanisms linking chronic physical conditions and perinatal mental illness, suggest that such interventions may positively impact the mental well-being of women and their families. However, it is important to note that the aRRs for the association between chronic physical conditions and perinatal mental illness ranged from 1.12 to 1.68 across the main analyses. The cost of developing targeted mental health interventions or screening for women with chronic physical conditions should be weighed against these relatively small effect sizes.

### Limitations

The Agency for Healthcare Research and Quality Chronic Condition Indicator for the ICD-10 is a comprehensive method by which to identify chronic physical conditions in administrative data. However, in Ontario, it can only be applied to acute healthcare encounters, because physician visits are coded using a 3-digit version of the ICD-9, which cannot distinguish between an acute versus a chronic disease. In our definition of chronic physical conditions, we likely captured those women with the most severe, complicated, or poorly managed conditions. Such individuals could be at greatest risk for perinatal mental illness. It is also possible that women with chronic physical conditions presenting in acute healthcare settings differ from those with chronic physical conditions more broadly on factors other than condition severity. For example, they could be more anxious or depressed but less likely to seek care for mental illness. The impact of misclassification on our RRs is therefore difficult to predict. Our definition may explain the higher proportion of women with a chronic physical condition living in rural areas, where emergency departments tend to be accessed for all levels of care. The generalizability of our cohort of women with chronic physical conditions to the target population should therefore be interpreted with caution. Finally, the algorithm classified chronic physical conditions by body system and not by each disease diagnosis, so specific details about how a condition influences mental health cannot be described.

Our definition of diagnosed perinatal mental illness required that a woman sought healthcare. Yet a significant proportion of women with perinatal mental illness never receive a diagnosis because of barriers to accessing care [42]. Hence, the current study may have underestimated the true rate of perinatal mental illness, especially in marginalized groups. Because of the truncated codes used for physician visits, we could not distinguish between depression and anxiety. However, research suggests that there is increased risk for both in association with chronic physical conditions [14]. We used liberal definitions of recent and remote history of mental health care, resulting in higher rates of these variables (25.7% and 47.9%, respectively) than in general-population self-reported surveys [43]. Finally, because our cohort was restricted to women without a recent history of mental illness, our results pertain to the development of new-onset mental illness or relapse in the perinatal period, with implications for prevention interventions. Future research could include all women, regardless of recent mental healthcare, to examine patterns of ongoing mental illness in the perinatal period associated with chronic physical conditions.

Finally, residual confounding may be a threat to the validity of our findings. Health administrative data were not collected for research purposes, and, as such, data on important confounders were missing. We did not have information on demographic variables such as ethnicity or individual-level measures of socioeconomic status such as income and education. We also did not have information on BMI, lifestyle factors such as diet and smoking, or social factors such as stressful life events or social support. These are all important confounders in the association between chronic physical conditions and perinatal mental illness [6]; however, we were unable to adjust for these variables in our multivariable models. Similarly, we were unable to adjust for shared genetic influences underlying both chronic physical conditions and perinatal mental illness [36]; this is an important area for future study.

### Conclusion

Findings from this large population-based study suggest that women with a chronic physical condition predating pregnancy are at heightened risk of mental illness in pregnancy and post partum. This risk remained higher for all specific mental illness diagnoses except self-harm and was largely consistent across chronic physical conditions affecting different body systems. These women may require targeted efforts to lower the severity of their condition and improve their coping strategies and supports in pregnancy and the postpartum period.

## Supporting information

S1 Checklist STROBE checklist. STROBE, Strengthening the Reporting of Observational Studies in Epidemiology.
(DOCX)

S1 Text Prospective analysis plan. (DOCX)

S1 Table Ascertainment of maternal chronic physical conditions using the Agency for Healthcare Research and Quality Chronic Condition Indicator for the ICD-10. ICD-10, International Statistical Classification of Diseases and Related Health Problems, 10th revision.
(DOCX)

S2 Table Type of perinatal mental illness and its method of ascertainment. (DOCX)

S3 Table Frequency of maternal chronic physical conditions, by body system. (DOCX)

S4 Table Risk of perinatal mental illness arising between conception and 1 year post partum, in relation to a woman having a chronic physical condition in the 24 months prior to conception, and further detailed by the type of “other” chronic physical condition. (DOCX)

S5 Table Risk of perinatal mental illness arising between conception and 1 year post partum, in relation to a woman having a chronic physical condition in the 24 months prior to conception, and further detailed by the type of chronic physical condition and timing of perinatal mental illness. (DOCX)

S6 Table Risk of a psychotic disorder or mood or anxiety disorder arising between conception and 1 year post partum, in relation to a woman having a chronic physical condition in the 24 months prior to conception, and further detailed by the type of chronic physical condition. (DOCX)

S7 Table Risk of a substance use disorder, self-harm, or other mental illness arising between conception and 1 year post partum, in relation to a woman having a chronic physical condition in the 24 months prior to conception, and further detailed by the type of chronic physical condition. (DOCX)

S8 Table Risk of perinatal mental illness requiring at least 2 physician visits between conception and 1 year post partum, in relation to a woman having a chronic physical condition in the 24 months prior to conception. (DOCX)

## Abbreviations

aRR: adjusted relative risk
CI: confidence interval
CPC: chronic physical condition
DSM: Diagnostic and Statistical Manual of Mental Disorders
ICD: International Statistical Classification of Diseases and Related Health Problems
OHIP: Ontario Health Insurance Plan
RR: relative risk
STROBE: Strengthening the Reporting of Observational Studies in Epidemiology

## References

[1] O'Hara MW, Wisner KL. Perinatal mental illness: Definition, description and aetiology. Best Pract Res Clin Obstet Gynecol. 2014;28(1): 3–12. 10.1016/j.bpobgyn.2013.09.002 PMC7077785 24140480

[2] Meltzer-Brody S, Stuebe A. The long-term psychiatric and medical prognosis of perinatal mental illness. Best Pract Res Clin Obstet Gynecol. 2014;8(1): 49–60. 10.1016/j.bpobgyn.2013.08.009 PMC3947371 24063973

[3] Deave T, Heron J, Evans J, Emond A. The impact of maternal depression in pregnancy on early child development. BJOG. 2008;115(8): 1043–1051. 10.1111/j.1471-0528.2008.01752.x 18651886

[4] Coates AO, Schaefer CA, Alexander JL. Detection of postpartum depression and anxiety in a large health plan. J Behav Health Serv Res. 2004;31(2): 117–133. 15255221 10.1007/BF02287376

[5] Howard LM, Piot P, Stein A. No health without perinatal mental health. Lancet. 2014;384(9956): 1723–1724. 10.1016/S0140-6736(14)62040-7 25455235

[6] Robertson E, Grace S, Wallington T, Stewart DE. Antenatal risk factors for postpartum depression: A synthesis of recent literature. Gen Hosp Psychiatry. 2004;26(4): 289–295. 10.1016/j.genhosppsych.2004.02.006 15234824

[7] Kersten I, Lange AE, Haas JP, Fusch C, Lode H, Hoffman W, et al Chronic diseases in pregnant women: Prevalence and birth outcomes based on the SNiP-study. BMC Pregnancy Childbirth. 2014;14: 75 10.1186/1471-2393-14-75 24552439 PMC3943445

[8] Secinti E, Thompson EJ, Richards M, Gaysina D. Research review: Childhood chronic physical illness and adult emotional health–a systematic review and meta-analysis. J Child Psychol Psychiatry. 2017;58(7): 753–769. 10.1111/jcpp.12727 28449285

[9] Ali S, Stone M, Peters J, Davies M, Khunti K. The prevalence of comorbid depression in adults with Type 2 diabetes: A systematic review and meta-analysis. Diabet Med. 2006;23(11): 1165–1173. 10.1111/j.1464-5491.2006.01943.x 17054590

[10] Li Z, Li Y, Chen L, Chen P, Hu Y. Prevalence of depression in patients with hypertension: A systematic review and mta-analysis. Medicine. 2015;94(31): e1317 10.1097/MD.0000000000001317 26252317 PMC4616591

[11] Scott KM, Von Korff M, Ormel J, Zhang MY, Bruffaerts R, Alonso J, et al Mental disorders among adults with asthma: Results from the World Mental Health Survey. Gen Hosp Psychiatry. 2007;29(2): 123–133. 10.1016/j.genhosppsych.2006.12.006 17336661 PMC1913936

[12] Chapman DP, Perry GS, Strine TW. The vital link between chronic disease and depressive disorders. Prev Chronic Dis. 2005;2(1): A14 15670467 PMC1323317

[13] Ehlert U, Gaab J, Heinrichs M. Psychoneuroendocrinological contributions to the etiology of depression, posttraumatic stress disorder, and stress-related bodily disorders: the role of the hypothalamus-pituitary-adrenal axis. Biol Psychol. 2001;57(1–3): 141–152. 11454437 10.1016/s0301-0511(01)00092-8

[14] Brown HK, Qazilbash A, Rahim N, Dennis CL, Vigod SN. Chronic medical conditions and perinatal mental illness: A systematic review and meta-analysis. Am J Epidemiol. 2018;187(9): 2060–2068. 10.1093/aje/kwy080 29635285

[15] Evers IM, de Valk HW, Visser GH. Risk of complications of pregnancy in women with type 1 diabetes: Nationwide prospective study in the Netherlands. BMJ. 2004;328(7445): 915 10.1136/bmj.38043.583160.EE 15066886 PMC390158

[16] Gilbert WM, Young AL, Danielsen B. Pregnancy outcomes in women with chronic hypertension: A population-based study. J Reprod Med. 2007;52(11): 1046–1051. 18161404

[17] Statistics Canada. Live births and fetal deaths (stillbirths) by place of birth (hospital and non-hospital), Canada, provinces and territories, annual, 2006. Ottawa, ON: Statistics Canada; 2006 Available from: http://www5.statcan.gc.ca/cansim/a26?lang=eng&id=1024516. [cited 2018 February 13].

[18] You JJ, Alter DA, Stukel TA, McDonald SD, Laupacis A, Liu Y, et al Proliferation of prenatal ultrasonography. CMAJ. 2010;182(2): 143–151. 10.1503/cmaj.090979 20048009 PMC2817321

[19] Williams JI, Young WA. Summary of studies on the quality of health care administrative databases in Canada In: Goel V, Williams JI, Anderson GM, Blackstien-Hirsch P, Fooks C, Naylor CD, editors. Patterns of Health Care in Ontario, The ICES Practice Atlas. 2nd ed Ottawa, ON: Canadian Medical Association; 1996 pp 339–345.

[20] Agency for Healthcare Research and Quality. Beta chronic condition indicator (CCI) for ICD-10-CM. Rockville, MD: Agency for Healthcare Research and Quality; 2018 Available from: https://www.hcup-us.ahrq.gov/toolssoftware/chronic_icd10/chronic_icd10.jsp. [cited 2018 March 19].

[21] Gill PJ, Saunders N, Gandhi S, Gonzalez A, Kurdyak P, Vigod S, et al Emergency departmetn as a first contact for mental health problems in children and youth. J Am Acad Child Adolesc Psychiatry 2017;56(6): 475–482. 10.1016/j.jaac.2017.03.012 28545752

[22] Steele LS, Glazier RH, Lin E, Evans M. Using administrative data to measure ambulatory mental health service provision in primary care. Med Care. 2004;42(10): 960–965. 15377928 10.1097/00005650-200410000-00004

[23] Urquia ML, Frank JW, Glazier RH, Moineddin R. Birth outcomes by neighbourhood income and recent immigration in Toronto. Health Rep. 2007;18: 21–30. 18074994

[24] Austin PC. Using the standardized difference to compare the prevalence of a binary variable between two groups in observational research. Comm Stat. 2009;38(6): 1228–1234.

[25] Zou GY. A modified Poisson regression approach to prospective studies with binary data. Am J Epidemiol. 2004;159(7): 702–706. 10.1093/aje/kwh090 15033648

[26] Zou GY. Extension of the modified Poisson regression model to prospective studies with correlated binary data. Stat Method Med Res. 2013;22(6): 661–670. 10.1177/0962280211427759 22072596

[27] Burgut F, Bener A, Ghuloum S, Sheikh J. A study of postpartum depression and maternal risk factors in Qatar. J Psychosom Obstet Gynecol. 2013;34(2): 90–97. 10.3109/0167482X.2013.786036 23701432

[28] Chaaya M, Campbell O, El Kak F, Shaar D, Harb H, Kaddour A. Postpartum depression: prevalence and determinants in Lebanon. Arch Womens Ment Health. 2002;5(2): 65–72. 10.1007/s00737-002-0140-8 12510201 PMC1457112

[29] Katon W, Russo J, Gavin A. Predictors of postpartum depression. J Womens Health. 2014; 23(9): 753–759. 10.1089/jwh.2014.4824 25121562

[30] Raisanen S, Lehto SM, Nielsen HS, Gissler M, Kramer MR, Heinonen S. Risk factors for and perinatal outcomes of major depression during pregnancy: A population-based analysis during 2002–2010 in Finland. BMJ Open. 2014;4(11): e004883 10.1136/bmjopen-2014-004883 25398675 PMC4244456

[31] Silverman ME, Reichenberg A, Savitz DA, Cnattingius S, Lichtenstein P, Hultman CM, et al The risk factors for postpartum depression: A population-based study. Depress Anxiety. 2017;34(2): 178–187. 10.1002/da.22597 28098957 PMC5462547

[32] Katon JG, Russo J, Gavin AR, Melville JL, Katon WJ. Diabetes and depression in pregnancy: Is there an association? J Womens Health. 2011;20(7): 983–989. 10.1089/jwh.2010.2662 PMC3130515 21668382

[33] Kozhimannil KB, Pereira MA, Harlow BL. Association between diabetes and perinatal depression among low-income mothers. JAMA. 2009;301(8): 842–847. 10.1001/jama.2009.201 19244191

[34] Miller ES, Peri MR, Gossett DR. The association between diabetes and postpartum depression. Arch Womens Ment Health. 2016;19(1): 183–186. 10.1007/s00737-015-0544-x 26184833

[35] Molyneaux E, Poston L, Ashurst-Williams S, Howard LM. Obesity and mental disorders during pregnancy and postpartum: a systematic review and meta-analysis. Obstet Gynecol. 2014;123(4): 857–867. 10.1097/AOG.0000000000000170 24785615 PMC4254698

[36] Kan C, Pedersen NL, Christensen K, Bornstein SR, Licinio J, MacCabe JH, et al Genetic overlap between type 2 diabetes and depression in Swedish and Danish twin registries. Mol Psychiatry. 2016;21: 903 10.1038/mp.2016.28 27021822 PMC5414070

[37] Gleicher N. Postpartum depression, an autoimmune disease? Autoimm Rev. 2007;6(8): 572–576. 10.1016/j.autrev.2007.04.002 17854751

[38] Van der Kooy K, van Hout H, Marwijk H, Marten H, Stehouwer C, Beekman A. Depression and the risk for cardiovascular diseases: Systematic review and meta analysis. Int J Geriatr Psychiatry. 2007;22(7): 613–626. 10.1002/gps.1723 17236251

[39] Katon WJ, Lin EH, Von Korff M, Ciechanowski P, Ludman EJ, Young B, et al Collaborative care for patients with depression and chronic illnesses. N Engl J Med. 2010;363(27): 2611–2620. 10.1056/NEJMoa1003955 21190455 PMC3312811

[40] Byatt N, Biebel K, Simas TAM, Sarvet B, Ravech M, Allison J, et al Improving perinatal depression care: The Massachusetts Child Psychiatry Access Project for Moms. Gen Hosp Psychiatry. 2016;40: 12–17. 10.1016/j.genhosppsych.2016.03.002 27079616

[41] Stephenson J, Heslehurst N, Hall J, Schenaker DAJM, Hutchinson J, Cade PE, et al Before the beginning: Nutrition and lifestyle in the preconception period and its importance for future heatlh. Lancet. 2018;391(10132):1830–1841. 10.1016/S0140-6736(18)30311-8 29673873 PMC6075697

[42] Tabb KM, Choi S, Pineros-Leano M, Meline B, McDonald HG, Kester R, et al Perinatal depression screening in a women, infants and children (WIC) program: Perception of feasibility and acceptability among a multidisciplinary staff. Gen Hosp Psychiatry. 2015;37(4): 305–309. 10.1016/j.genhosppsych.2015.03.008 25858684

[43] Statistics Canada. Mental and substance use disorders in Canada. Ottawa, ON: Statistics Canada; 2006 Available from: https://www150.statcan.gc.ca/n1/pub/82-624-x/2013001/article/11855-eng.htm. [cited 2018 Jun 15].

