# Supplementary material for: Chronic physical conditions and risk for perinatal mental illness: A population-based retrospective cohort study
Source: PLoS Med. 2019 Aug 26;16(8):e1002864. doi: 10.1371/journal.pmed.1002864 (PMC6709891; doi:10.1371/journal.pmed.1002864)
Supplement: S1 Text — (DOCX) [file pmed.1002864.s002.docx]

| Project InitiationThis Section must be Completed Prior to Project Dataset(s) Creation | | | | | |
| --- | --- | --- | --- | --- | --- |
| **Project Title:** | Chronic Medical Conditions and Perinatal Mental Illness | | | | |
| **Project TRIM number:** | 2018 0990 014 000 | | | | |
| **Research Program:** | MHA | | | | |
| **Site:** | ICES Central | | | | |
| **Project Objectives:** | *Insert Project Objectives as listed in the approved ICES Project PIA* | | | | |
|  | To determine the independent effect of CMC on the risk for perinatal mental illness, including prenatal and postpartum mental illness. | | | | |
| **ICES Project PIA Initial Approval Date:** | *The ICES Employee or agent who is responsible for creating the Project Dataset(s) is responsible for ensuring there is an approved ICES Project PIA and verifying the date of approval prior to creating the Project Dataset(s)* | | | | |
|  | 2017-Jul-17 | | | | |
| **Principal Investigator (PI):** | Hilary Brown | | | | |
| **Check the applicable box if the PI is an ICES Student/Trainee** | ICES Student  ICES Fellow  ICES Post-Doctoral Trainee  Visiting Scholar | | | | |
| **Responsible ICES Scientist:** | *Name the Responsible ICES Scientist if the PI is not a Full Status ICES Scientist* | | | | |
|  |  | | | | |
| **Project Team Member(s) Responsible for Project Dataset Creation and/or Statistical Analysis and date joined (list all):** | *All person(s) (ICES Analyst, Appointed Analyst, Analytic Epidemiologist, PI, and/or Student) responsible for creating the Project Dataset(s) and/or statistical analysis on the Research Analytics Environment (RAE) and the date they joined the project must be recorded* | | | | |
|  | Hilary Brown | | | 2017-Jul-07 | |
| **Other ICES Project Team Members and date joined (list all):** | *All other Research Project Team Members (e.g., Research Administrative Assistants, Research Assistants, Project Managers, Epidemiologists) and the date they joined the project must be recorded* | | | | |
|  | Cindy-Lee Dennis  Astrid Guttmann  Joel Ray  Simone Vigod  Andrew Wilton | | | 2017-SEP-19 | |
| **Confirmation that DCP is consistent with Project Objectives:** | *The following individuals must confirm that the ICES Data provided for in this DCP is relevant (e.g., with respect to cohort, timeframe, and variables) and required to achieve the Project Objectives stated in the ICES Project PIA prior to initial Project Dataset creation: 1) PI; 2) Responsible ICES Scientist if the PI is not a Full Status ICES Scientist, or a second ICES Scientist or the Scientific Program Lead if the PI is creating both the DCP and the Project Dataset[s]; 3) ICES Research and Analysis Staff creating the DCP; and 4) ICES Analytic Staff (ICES Employee or agent responsible for creating the Project Dataset[s]). This may be delegated either verbally or via e-mail.* | | | | |
|  | ***Principal Investigator*** | |  | | 2017-Jul-26 |
|  | ***Responsible ICES Scientist or Second ICES Scientist/Lead*** | |  | yyyy-mon-dd | |
|  | ***ICES Research and Analysis Staff Creating the DCP*** | |  | yyyy-mon-dd | |
|  | ***ICES Analytic Staff*** | |  | yyyy-mon-dd | |
| **Designated ICES Research and Analysis Staff accountable for Project Documentation:** | *The person named (ICES staff) is accountable for ensuring that the approved ICES Project PIA, ICES Project PIA Amendments, and DCP are saved on the T Drive, ensuring ICES Project PIA Amendments are submitted as required, ensuring DCP Amendments are documented, and sharing the final DCP with the PI/Responsible ICES Scientist at project completion* | | | | |
|  | Andrew Wilton | | | | |
| **DCP Creation Date and Author:** | *Date DCP was finalized prior to Project Dataset(s) creation* | *Name of person who created the DCP* | | | |
|  | ***Date*** | ***Name*** | | | |
|  | 2017-Jul-26 | Hilary Brown | | | |

| ICES DataThis Section must be Completed Prior to Project Dataset(s) Creation | |
| --- | --- |
| *The ICES Employee or agent who is responsible for creating the Project Dataset(s) must ensure that this list includes only data listed in the ICES Project PIA*  *Changes to this list after initial ICES Project PIA approval require an ICES Project PIA Amendment* | *Mandatory for all datasets that are available by individual year* |
| ***General Use Datasets – Health Services*** | ***Years (where applicable)*** |
| CIHI DAD | April 1, 2000 to December 31, 2016 |
| CIHI SDS | April 1, 2000 to December 31, 2016 |
| NACRS | April 1, 2000 to December 31, 2016 |
| OMHRS | April 1, 2000 to December 31, 2016 |
| OHIP | April 1, 2000 to December 31, 2016 |
| CONTACT | April 1, 2000 to December 31, 2016 |
| ***General Use Datasets – Care Providers*** |  |
| IPDB | April 1, 2013 to March 31, 2015 |
| See list |  |
| ***General Use Datasets – Population*** |  |
| RPDB | April 1, 2005 to March 31, 2015 |
| CENSUS | April 1, 2005 to March 31, 2015 |
| ***General Use Datasets – Coding/Geography*** |  |
| PCCF | April 1, 2005 to March 31, 2015 |
| See list |  |
| ***General Use Datasets - Facilities*** |  |
| See list |  |
| ***General Use Datasets - Other*** |  |
| MOMBABY | April 1, 2015 to March 31, 2015 |
| CAPE | April 1, 2013 to March 31 2015 |
| ***Controlled Use Datasets*** |  |
| See list |  |
| ***Other Datasets*** |  |

| Project Amendments and Reconciliation | | | |
| --- | --- | --- | --- |
| **ICES Project PIA Amendment History (add additional rows as needed):** | *Privacy approval date* | *Person who submitted amendment* | *Note that any changes to the list of ICES Data or Project Objectives require an ICES Project PIA Amendment* |
|  | ***Date*** | ***Name*** | ***Amendment*** |
|  | yyyy-mon-dd |  |  |
| **DCP Amendment History (add additional rows as needed):** | *Date DCP amended* | *Person who made the DCP amendment* | *Note that any DCP amendments involving changes to the list of ICES Data or Project Objectives require an ICES Project PIA Amendment* |
|  | ***Date*** | ***Name*** | ***Amendment*** |
|  | 2019-May-17 | Hilary Brown | Added non-mutually exclusive ‘type of perinatal mental illness’ variables |

| Project Cohort | | |
| --- | --- | --- |
| **Study Design** | Cohort study  Matched cohort study  Case-control study  Cross-sectional study  Other (specify): | |
| **Index Event / Inclusion Criteria** | All women in Ontario hospitalized for one or more obstetrical deliveries of a singleton liveborn infant after 20 weeks gestational age with a conception date between April 1, 2005 and March 31, 2015. We will identify obstetrical deliveries using the MOMBABY datafile. The index event will be the estimated date of conception (see details below). | |
| **Index Date (cohort entry)** | The **estimated date of conception** is derived from the MOMBABY dataset using the variables b_bdate and m_gestwks_del. When baby_bdate is missing, use mother’s admission date as the delivery date. Calculate as:   - b_ bdate -m_gestwks_del OR - admdate-m_gestwks_del (use when baby_bday variable is missing) | |
| **Estimated Size of Cohort**  **(if known)** |  | |
| **Exclusions (in order)** | *Step* | Description |
|  | 1 | Invalid IKN for mother (m_valikn ≠ V in MOMBABY) or invalid IKN for baby (b_valikn ≠ V) |
|  | 2 | Ineligible for OHIP: use %getelig macro and ELIG variable in CONTACT file to check continuous OHIP eligibility, from conception minus 730 days to delivery plus 365 days (Check numbers) |
|  | 3 | Missing gender or gender ≠ F for mother |
|  | 4 | Multiple birth (MOMBABY b_multibirth=T) or stillbirth (b_stillbirth=T) |
|  | 5 | Gestational age at delivery less than 20 weeks (140 days) |
|  | 6 | Maternal death date prior to delivery date (dthdate<delivery date) |
|  | 7 | Women younger than 15 years and older than 49 years at conception date (m_age variable in MOMBABY) |
|  | 8 | Women with a history of mental illness in the 2 years before the index pregnancy conception date as indicated by ≥ 1 physician visits, hospitalizations, or emergency department visits:   - OHIP: (1) with a general practitioner/family physician [SPEC=00]: (a) a general service code (A001, A003-A008, A888, A901, A905) with a mental health diagnostic code (295-298, 300-304, 309, 311) or (b) a mental health service code (K005, K007, K623) for diagnostic codes 295-298, 300-304, 309, 311 **OR** (2) any visit with a psychiatrist [SPEC=19] EXCEPT for diagnostic codes 897-902, 904-906, or 909 - LOCATION: O, L, H - Exclude all G codes (lab codes) - Visit = 1 claim/IKN/physnum/servdate - CIHI-DAD/NACRS: (1) DX10CODE1 = F04-F99 **OR** (2) DX10CODE2 to DX10CODE10 = X60-X84, Y10-Y19 AND DX10CODE1 not equal to F04-F99 - Include visits/admissions with suspect diagnoses (suspect=T) and ACUTE=F. - OMHRS: Any (including missing diagnoses; excluding 290.x and 294.x) - If variable AXIS1_DSM4CODE_DISCH1 is complete (i.e., listed diagnosis from above present), use AXIS1_DSM4CODE_DISCH1. If not, use PROVDX1 (except PROVDX=2 Delirium, Dementia, Amnestic, Other Cog Disorder). |
|  | 9 | Non-residents of Ontario (cnty in ‘OP,’00’) |
|  | 10 | Mother and baby with same IKN |

| Project Time Frame Definitions | | |
| --- | --- | --- |
| Look-back Window  Observation Window  (in which to look for outcomes)  **Index Event Date**  Accrual Window  Max Follow-up Date | |  |
| **Accrual Start/End Dates** | April 1, 2005 to March 31, 2015 |  |
| **Max Follow-up Date** | December 31, 2016 |  |
| **When does observation window terminate?** | 365 days after delivery date so that everyone is followed for the full pregnancy plus 12 months postpartum |  |
| **Lookback Window(s)** | From DATE OF COHORT ENTRY: April 1, 2000 (5 years) for most exposures. See below for details. |  |

| Variable Definitions (add additional rows as needed) | | |
| --- | --- | --- |
| **Main Exposure or Risk Factor** | **Definition 1a**: Any CMC vs. none as per AHRQ list, where presence of a CMC is defined as ≥ 1 hospital encounters (CIHI-DAD, SDS, NACRS using any dx10code except dxtype=3) in the 2 years prior to the index pregnancy conception date  <https://www.hcup-us.ahrq.gov/toolssoftware/chronic_icd10/chronic_icd10.jsp>  **Definition 1b**: Any CMC *by body system* (separate variables, with each CMC group vs. no CMC group), as per AHRQ list, where presence of a CMC is defined as ≥ 1 hospital encounters (CIHI-DAD, SDS, NACRS using any dx10code except dxtype=3) in the 2 years prior to the index pregnancy conception date   1. Infections and parasitic disease = 1 2. Neoplasm = 2 3. Endocrine, nutritional, and metabolic diseases and immunity disorders = 3 4. Diseases of the blood and blood-forming organs = 4 5. Diseases of the nervous system and sense organs = 6 6. Diseases of the circulatory system = 7 7. Diseases of the respiratory system = 8 8. Diseases of the digestive system = 9 9. Diseases of the genitourinary system = 10 10. Diseases of the skin and subcutaneous tissue = 12 11. Diseases of the musculoskeletal system = 13 12. Congenital anomalies = 14 13. Injury and poisoning = 17 14. Factors influencing health status and contact with health services = 18 15. Multi-CMC = two or more of the above, 1 of the above, or none |  |
| **Comparison group**: | Women without CMC as defined above. Note that each obstetrical delivery should be examined separately. A woman could be considered ‘unexposed’ in one pregnancy but ‘exposed’ in the other. We will control for clustering within women in the analysis. |  |
| **Exposure group sensitivity analysis definition** |  |  |
| **Primary Outcome Definition** | **Perinatal mental illness**: ≥ 1 physician visits, hospitalizations, or emergency department visits between the conception date and 365 days after the delivery date. For hospitalizations, use admission date to establish timing:   - OHIP: (1) with a general practitioner/family physician [SPEC=00]: (a) a general service code (A001, A003-A008, A888, A901, A905) with a mental health diagnostic code (295-298, 300-304, 309, 311) or (b) a mental health service code (K005, K007, K623) for diagnostic codes 295-298, 300-304, 309, 3011 **OR** (2) any visit with a psychiatrist [SPEC=19] EXCEPT for diagnostic codes 897-902, 904-906, or 909   - LOCATION: O, L, H   - Exclude all G codes (lab codes)   - Visit = 1 claim/IKN/physnum/servdate - CIHI-DAD/NACRS: (1) DX10CODE1 = F04-F99 OR (2) DX10CODE2 to DX10CODE10 = X60-X84, Y10-Y19 AND DX10CODE1 not equal to F04-F99   - Include visits/admissions with suspect diagnoses (suspect=T) and ACUTE=F. - OMHRS: Any (including missing diagnoses; excluding 290.x and 294.x)   - If variable AXIS1_DSM4CODE_DISCH1 is complete (i.e., listed diagnosis from above present), use AXIS1_DSM4CODE_DISCH1. If not, use PROVDX1 (except PROVDX=2 Delirium, Dementia, Amnestic, Other Cog Disorder). |  |
| **Secondary Outcome Definition(s)** | **Timing of perinatal mental illness:**   1. Prenatal: ≥ 1 physician visits, hospitalizations, or emergency department visits with a diagnostic code for a mental illness (**as defined above**) where the encounter/admission date of the first encounter occurs between the conception date and the day before the delivery date 2. Postnatal: ≥ 1 physician visits, hospitalizations, or emergency department visits with a diagnostic code for a mental illness (**as defined above**) where the encounter/admission date of the first encounter occurs between the delivery date and 365 days after this date   **Type of perinatal mental illness:** For each of the following, use ≥ 1 physician visits hospitalizations, or emergency department visits between the conception date and 365 days after the date of delivery. For hospitalizations, use admission date to establish timing. As above:   - For physician visits, include visits to a general practitioner/family physician [SPEC=00] or psychiatrist [SPEC=19] as above, but be sure to differentiate type of perinatal mental illness using DXCODES below.   - LOCATION: O, L, H; Exclude all G codes (lab codes); Visit = 1 claim/IKN/physnum/servdate. - For hospitalizations and emergency department visits, include visits/admissions with suspect diagnoses (suspect=T) and ACUTE=F. - For OMHRS admissions, if variable AXIS1_DSM4CODE_DISCH1 is complete (i.e., listed diagnosis from above present), use AXIS1_DSM4CODE_DISCH1. If not, use PROVDX1 (except PROVDX=2 Delirium, Dementia, Amnestic, Other Cog Disorder).   Type of perinatal mental illness is then coded as follows based on first recorded health care encounter:   1. Psychotic disorder:    1. OHIP: (1) with a general practitioner/family physician [SPEC=00]: (a) a general service code (A001, A003-A008, A888, A901, A905) for diagnostic codes 295, 297, 298 or (b) a mental health service code (K005, K007, K623) for diagnostic codes 295, 296, 297, 298 **OR** (2) a visit with a psychiatrist [SPEC=19] for diagnostic codes 295, 296, 297, 298    2. CIHI-DAD/NACRS: DX10CODE1 F20 (excluding F20.4), F22-F25, F28-F29, F53.1    3. OMHRS: DSM-IV 295.x (all 295 codes), 297.x (all 297 codes), 298.x (all 298 codes); PROVDX1: 5 2. Mood / anxiety disorder:    1. OHIP: (1) with a general practitioner/family physician [SPEC=00]: (a) a general service code (A001, A003-A008, A888, A901, A905) for diagnostic codes 300, 309, 311 or (b) a mental health service code (K005, K007, K623) for diagnostic codes 300, 309, 311 **OR** (2) a visit with a psychiatrist [SPEC=19] for diagnostic codes 300, 309, 311    2. CIHI-DAD/NACRS: DX10CODE1 F30-F34, F38-F43, F48.8, F48.9, F53.0    3. OMHRS: DSM-IV 296.x (all 296 codes), 300, 300.0x, 300.2x, 300.3x, 300.4x, 301.13, 308.3x, 309.0x, 309.24, 309.28, 309.3x, 309.4x, 309.8x, 309.9x, PROVDX1: 6, 7, 15 3. Substance use disorder:    1. OHIP: (1) with a general practitioner/family physician [SPEC=00]: (a) a general service code (A001, A003-A008, A888, A901, A905) for diagnostic codes 303, 304 or (b) a mental health service code (K005, K007, K623) for diagnostic codes 303, 304 **OR** (2) a visit with a psychiatrist [SPEC=19] for diagnostic codes 303, 304    2. CIHI-DAD/NACRS: DX10CODE1 F10-F19, F55    3. OMHRS: DSM-IV 291.x (all 291 codes, excluding 291.82), 292.x (all 292 codes, excluding 292.85), 303.x (all 303 codes), 304.x (all 304 codes), 305.x (all 305 codes), PROVDX: 4 4. Self-harm:    1. CIHI-DAD/NACRS: X60-X84, Y10-Y19, Y28 listed in DX10CODE2 to DX10CODE10 5. Other:    1. OHIP: (1) with a general practitioner/family physician [SPEC=00]: (a) a general service code (A001, A003-A008, A888, A901, A905) for diagnostic codes 301, 302, 306 or (b) a mental health service code (K005, K007, K623) for diagnostic codes 301, 302, 306 **OR** (2) a visit with a psychiatrist [SPEC=19] for diagnostic codes 301, 302, 306    2. CIHI-DAD/NACRS: DX10CODE1 F21, F60-F62, F68, F69    3. OMHRS: DSM-IV 300.16, 300.19, 301.x (all 301 codes excluding 301.1x), PROVDX: 1, 16.   ***Please also create psychotic disorder, mood or anxiety disorder, substance use disorder, self-harm, and other mental illness variables based on ANY encounter in follow-up period (not just the first encounter). These will not be mutually exclusive.** |  |
| **Baseline Characteristics** | **Age**: defined on conception date (RPDB), in 5-year categories: 15-19, 20-24, 25-29, 30-34, 35-39, 40-44, and 45-49 years and as mean (SD)  **Parity**: defined on conception date (MOMBABY, CIHI-DAD from 2002 onwards)   - link m_key in MOMBABY to key variable in DADKEY database and extract prevbirth variable from CIHI-DAD - Categorize this variable into:   - Primiparous (no previous deliveries prevbirth=0)   - Multiparous (1 or more previous deliveries prevbirth>=1)   - Missing (missing value prevbirth=missing)   **Rural/remote residence**: Rural residence based on postal code, as of the index pregnancy conception date   - List as rural (< 10,000) or urban   **Low income status**: Neighbourhood income quintile based on postal code, as of the index pregnancy conception date (RPDB incquint variable)   - List by neighbourhood income quintile for descriptive purposes and for regression models categorize quintiles 1 or 2 for low income and quintile 3, 4, or 5 for moderate/high income   **Remote history of mental illness**: ≥ 1 physician visits with a psychiatrist [SPEC=19] or general practitioner/family physician [SPEC=00], hospitalizations, or emergency department visits (without admission) from database inception to 2 years prior to the index pregnancy coneption date. For hospitalizations, use admission date to establish timing:   - OHIP: (1) with a general practitioner/family physician [SPEC=00]: (a) a general service code (A001, A003-A008, A888, A901, A905) with a mental health diagnostic code (295-298, 300-304, 309, 311) or (b) a mental health service code (K005, K007, K623) for diagnostic codes 295-298, 300-304, 309, 3011 **OR** (2) any visit with a psychiatrist [SPEC=19] EXCEPT for diagnostic codes 897-902, 904-906, or 909   - LOCATION: O, L, H   - Exclude all G codes (lab codes)   - Visit = 1 claim/IKN/physnum/servdate - CIHI-DAD/NACRS: ICD-9: (1) DXCODE1 = 290-319 OR (2) ECODE1 or ECODE2 = E950-E958 or E980-E988 AND DX10CODE1 not equal to 290-319; ICD-10: (1) DX10CODE1 = F04-F99 OR (2) DX10CODE2 to DX10CODE10 = X60-X84, Y10-Y19 AND DX10CODE1 not equal to F04-F99   - Include visits/admissions with suspect diagnoses (suspect=T) and ACUTE=F. - OMHRS: Any (including missing diagnoses; excluding 290.x and 294.x)   If variable AXIS1_DSM4CODE_DISCH1 is complete (i.e., listed diagnosis from above present), use AXIS1_DSM4CODE_DISCH1. If not, use PROVDX1 (except PROVDX=2 Delirium, Dementia, Amnestic, Other Cog Disorder). |  |
| **Other Variables** |  |  |

| Analysis Plan and Dummy Tables (expand/modify as needed) | | | | |
| --- | --- | --- | --- | --- |
| **Descriptive Tables:** | | | | |
| **Table 1. Baseline characteristics of women with and without CMC** | | | | |
| **Table 2. Unadjusted and adjusted RR for relationship between CMC and perinatal mental illness** | | | | |
| **Table 3, etc.: Sensitivity analyses** | | | | |
|  | | | | |
| **Statistical Model(s)** | | | | |
| **Type of model** | | Modified Poisson regression to directly estimate relative risk. Use generalized estimating equations (GEE) to account for multiple deliveries to the mother during the study period, *as per Zou et al., Am J Epidemiol 2015;159:702-706*. | | |
| **Primary independent variable** | | 1. Any CMC vs. no CMC 2. CMC by body system vs. no CMC 3. Multi-CMC (by body system) and 1 CMC vs. no CMC | | |
| **Dependent variable** | | 1. Any perinatal mental illness vs. no perinatal mental illness 2. Any prenatal mental illness vs. no prenatal mental illness 3. Any postpartum mental illness vs. no postpartum mental illness 4. Any perinatal psychotic disorder vs. no perinatal psychotic disorder 5. Any perinatal mood/anxiety disorder vs. no perinatal mood/anxiety disorder 6. Any perinatal substance disorder vs. no perinatal substance disorder 7. Any perinatal self-harm vs. no perinatal self-harm 8. Any other perinatal mental illness vs. no other perinatal mental illness | | |
| **Covariates** | | Maternal age, parity, low income status, rural/remote residence, remote history of mental illness | | |
| **Report** | | Unadjusted and Adjusted RR (95% CI) | | |
|  | |  | | |
| Quality Assurance Activities | | | | |
| **RAE Directory of SAS Programs** |  | | | |
| **RAE Directory of Final Dataset(s)** | *The* *final analytic dataset for each cohort includes all the data required to create the baseline tables and run all the models. It should include all covariates for all models such as patient risk factors, hospital characteristics, physician characteristics, exposure measures (continuous, categorical) and outcomes. It should include covariates that were considered but didn’t make the final cut. This would permit an analyst to easily re-run the models in the future.* | | | |
|  |  | | | |
| **RAE README file available:** Yes No | | | | |
| **Date results of quality assurance tools for final dataset shared with project team (where applicable):** | | | |  |
|  | | | **%assign** | yyyy-mon-dd |
|  | | | **%evolution** | yyyy-mon-dd |
|  | | | **%dinexplore** | yyyy-mon-dd |
|  | | | **%track / %exclude** | yyyy-mon-dd |
|  | | | **%codebook** | yyyy-mon-dd |
| **Additional comments:** | | |  | |
