# Supplementary material for: Chronic physical conditions and risk for perinatal mental illness: A population-based retrospective cohort study
Source: PLoS Med. 2019 Aug 26;16(8):e1002864. doi: 10.1371/journal.pmed.1002864 (PMC6709891; doi:10.1371/journal.pmed.1002864)
Supplement: S1 Table — ICD-10, International Statistical Classification of Diseases and Related Health Problems, 10th revision. (DOCX) [file pmed.1002864.s003.docx]

**S1 Table. Ascertainment of maternal chronic physical conditions using the Agency for Healthcare Research and Quality Chronic Condition Indicator for the ICD-10.**

| **Body system** | **Major examples of included conditions^a^** |
| --- | --- |
| Endocrine, nutritional, and metabolic diseases and immunity disorders | Goitre  Obesity  Ovarian dysfunction  Type 1 diabetes mellitus  Type 2 diabetes mellitus |
| Diseases of the circulatory system | Atrial fibrillation  Cardiac arrhythmia  Hypertension  Noninfective disorders of the lymphatic vessels and lymph nodes  Paroxysmal tachycardia |
| Diseases of the respiratory system | Asthma  Chronic diseases of the tonsils and adenoids  Chronic obstructive pulmonary disease  Chronic sinusitis, rhinitis, nasopharyngitis, or pharyngitis  Vasomotor and allergic rhinitis |
| Diseases of the musculoskeletal system | Gout  Internal derangement of the knee  Intervertebral disc disorders  Osteoarthritis  Rheumatoid arthritis |
| Diseases of the nervous system and sense organs | Epilepsy  Migraine  Mononeuropathy  Multiple sclerosis  Otitis externa (excluding acute) |
| Diseases of the digestive system | Crohn’s disease  Chronic gastritis  Gastroesophageal reflux disease  Irritable bowel syndrome  Ulcerative colitis |
| Diseases of the genitourinary system | Abnormal uterine and vaginal bleeding  Chronic salpingitis and oophoritis  Chronic cystitis  Endometriosis  Infertility |
| Diseases of the skin and subcutaneous system | Chronic ulcer of the skin  Lupus erythematosus  Psoriasis  Rosacea |
| Diseases of the blood and blood-forming organs | Agranulocytosis  Coagulation defects  Iron deficiency anaemia  Sickle cell disorders  Thrombocytopenia |
| Neoplasms | Malignant melanoma  Malignant neoplasm of the breast  Malignant neoplasm of the cervix  Malignant neoplasm of the lymph nodes  Malignant neoplasm of the thyroid gland |
| Infections and parasitic disease | Chronic viral hepatitis  Herpes simplex infection  Human immunodeficiency virus |
| Congenital anomalies | Congenital malformations of the breast  Congenital malformations of the cardiac septa  Congenital malformations of the genitals  Congenital malformations of the ovaries, fallopian tubes, and broad ligaments  Congenital malformations of the uterus and cervix |
| Injuries | Spinal cord injury  Traumatic amputation of a lower limb  Traumatic amputation of an upper limb |

^a^ The Agency for Healthcare Research and Quality Chronic Condition Indicator for the ICD-10 organizes chronic physical conditions by ICD-10 chapter and includes all conditions that last 12 months or longer and (1) limit independent living, social interactions, or self-care or (2) require ongoing intervention with medical services, products, or special equipment [1]. The examples provided herein are the most frequent conditions within each category and are not an exhaustive list.

**References**

1. Agency for Healthcare Research and Quality. Beta chronic condition indicator (CCI) for ICD-10-CM. Rockville, MD: Agency for Healthcare Research and Quality; 2018. Available: https://www.hcup-us.ahrq.gov/toolssoftware/chronic_icd10/chronic_icd10.jsp (accessed 2018 Mar. 19)
