# Supplementary material for: Chronic physical conditions and risk for perinatal mental illness: A population-based retrospective cohort study
Source: PLoS Med. 2019 Aug 26;16(8):e1002864. doi: 10.1371/journal.pmed.1002864 (PMC6709891; doi:10.1371/journal.pmed.1002864)
Supplement: S2 Table — (DOCX) [file pmed.1002864.s004.docx]

**S2 Table. Type of perinatal mental illness and its method of ascertainment.**

| **Type of mental illness** | **Outpatient physician diagnostic codes and service codes** | **ICD-10 codes for hospitalizations and emergency department visits** | **DSM-IV codes for psychiatric hospitalizations** |
| --- | --- | --- | --- |
| Psychotic disorders | Service codes A001, A003-A008, A888, A901, A905 (with a family physician) or K005, K007, K623 (with a psychiatrist) with diagnostic codes 295-298 | F20 (excluding F20.4), F22-F25, F28-F29, F53.1 | 295, 297, 298; PROVDX: 5 |
| Mood and anxiety disorders | Service codes A001, A003-A008, A888, A901, A905 (with a family physician) or K005, K007, K623 (with a psychiatrist) with diagnostic codes 300, 309, 311 | F30-F34, F38-F43, F48.8, F48.9, F53.0 | 296, 300, 300.0x, 300.2x, 300.3x, 300.4x, 301.13, 308.3x, 309.0x, 309.24, 309.28, 309.3x, 309.4x, 309.8x, 309.9x, PROVDX: 6, 7, 15 |
| Substance use disorders | Service codes A001, A003-A008, A888, A901, A905 (with a family physician) or K005, K007, K623 (with a psychiatrist) with diagnostic codes 303, 304 | F10-F19, F55 | All 291 codes except 291.82, all 292 codes except 292.85, 303, 304, 305, PROVDX: 4 |
| Other mental illness | Service codes A001, A003-A008, A888, A901, A905 (with a family physician) or K005, K007, K623 (with a psychiatrist) with diagnostic codes 301, 302, 306 | F21, F60-F62, F68, F69 | 300.16, 300.19, all 301 codes except 301.1, PROVDX: 1, 16 |
| Self-harm | --- | X60-X84, Y10-Y19, Y28 with or without F04-F99 as the primary diagnosis | --- |

Abbreviation: ICD-10 International Statistical Classification of Diseases and Health-Related Conditions, tenth revision; DSM-IV Diagnostic and Statistical Manual of Mental Disorders, 4^th^ revision.
