# Supplementary material for: Chronic physical conditions and risk for perinatal mental illness: A population-based retrospective cohort study
Source: PLoS Med. 2019 Aug 26;16(8):e1002864. doi: 10.1371/journal.pmed.1002864 (PMC6709891; doi:10.1371/journal.pmed.1002864)
Supplement: S3 Table — (DOCX) [file pmed.1002864.s005.docx]

**S3 Table. Frequency of maternal chronic physical conditions, by body system.**

| **Body system affected by maternal chronic physical conditions** | **Number (%)^a^** |
| --- | --- |
| Endocrine, nutritional, and metabolic diseases and immunity disorders | 5,014 (6.5) |
| Diseases of the circulatory system | 2,496 (3.2) |
| Diseases of the respiratory system | 12,442 (16.1) |
| Diseases of the musculoskeletal system | 3,994 (5.2) |
| Diseases of the nervous system and sense organs | 12,026 (15.5) |
| Diseases of the digestive system | 10,352 (13.4) |
| Diseases of the genitourinary system | 34,442 (44.5) |
| Diseases of the skin and subcutaneous tissue | 436 (0.56) |
| Diseases of the blood and blood-forming organs | 772 (1.0) |
| Neoplasms | 1,047 (1.4) |
| Infections and parasitic diseases | 670 (0.84) |
| Congenital anomalies | 2,376 (3.1) |
| Injury and poisoning | 160 (0.21) |

^a^ Percentage of those with chronic physical conditions who had a condition in this body system. The total percentage adds up to more than 100%, as an individual woman may have chronic physical conditions affecting more than one body system.
