# Supplementary material for: Chronic physical conditions and risk for perinatal mental illness: A population-based retrospective cohort study
Source: PLoS Med. 2019 Aug 26;16(8):e1002864. doi: 10.1371/journal.pmed.1002864 (PMC6709891; doi:10.1371/journal.pmed.1002864)
Supplement: S4 Table — (DOCX) [file pmed.1002864.s006.docx]

**S4 Table. Risk of perinatal mental illness arising between conception and 1 year postpartum, in relation to a woman having a chronic physical condition in the 24 months prior to conception, and further detailed by the type of “other” chronic physical condition.**

| **Variable** | **Number (%) with outcome** | **Unadjusted** | | **Adjusted^a^** | |
| --- | --- | --- | --- | --- | --- |
|  |  | **Relative risk (95% CI)** | **p-value** | **Relative risk (95% CI)** | **p-value** |
| Diseases of the skin and subcutaneous tissue |  |  |  |  |  |
| Absent (N = 857,568) | 137,448 (16.0) | 1.00 (referent) |  | 1.00 (referent) |  |
| Present (N = 436) | 80 (18.4) | 1.12 (0.92-1.37) | .27 | 0.97 (0.80-1.19) | .79 |
| Diseases of the blood and blood-forming organs |  |  |  |  |  |
| Absent (N = 857,232) | 137,372 (16.0) | 1.00 (referent) |  | 1.00 (referent) |  |
| Present (N = 772) | 156 (20.2) | 1.25 (1.08-1.44) | .002 | 1.11 (0.97-1.28) | .13 |
| Neoplasms |  |  |  |  |  |
| Absent (N = 856,957) | 137,346 (16.0) | 1.00 (referent) |  | 1.00 (referent) |  |
| Present (N = 1,047) | 182 (17.4) | 1.07 (0.94-1.22) | .31 | 1.03 (0.90-1.18) | .65 |
| Congenital anomalies |  |  |  |  |  |
| Absent (N = 855,628) | 137,101 (16.0) | 1.00 (referent) |  | 1.00 (referent) |  |
| Present (N = 2,376) | 427 (18.0) | 1.11 (1.02-1.21) | .01 | 1.01 (0.92-1.10) | .89 |
| Injury and poisoning |  |  |  |  |  |
| Absent (N = 857,844) | 137,505 (16.0) | 1.00 (referent) |  | 1.00 (referent) |  |
| Present (N = 160) | 23 (14.4) | 0.92 (0.64-1.31) | .63 | 0.81 (0.56-1.18) | .28 |

Note: These data show the breakdown of results for the “other” chronic physical condition category in Figure 2.

^a^ Adjusted for age, parity, rural residence, neighbourhood income quintile, remote history of mental health care, and other chronic physical conditions.
