# Supplementary material for: Chronic physical conditions and risk for perinatal mental illness: A population-based retrospective cohort study
Source: PLoS Med. 2019 Aug 26;16(8):e1002864. doi: 10.1371/journal.pmed.1002864 (PMC6709891; doi:10.1371/journal.pmed.1002864)
Supplement: S5 Table — (DOCX) [file pmed.1002864.s007.docx]

**S5 Table.** **Risk of perinatal mental illness arising between conception and 1 year postpartum, in relation to a woman having a chronic physical condition in the 24 months prior to conception, and further detailed by the type of chronic physical condition and timing of perinatal mental illness.**

|  | **Prenatal mental illness** | | | **Postpartum mental illness** | | |
| --- | --- | --- | --- | --- | --- | --- |
| **Variable** | **Number (%) with outcome** | **Unadjusted relative risk (95% CI)** | **Adjusted relative risk (95% CI)^a^** | **Number (%) with outcome** | **Unadjusted relative risk (95% CI)** | **Adjusted relative risk (95% CI)^a^** |
| Endocrine and metabolic disorders |  |  |  |  |  |  |
| Absent (N = 852,990) | 53,596 (6.3) | 1.00 (referent) | 1.00 (referent) | 82,908 (9.7) | 1.00 (referent) | 1.00 (referent) |
| Present (N = 5,014) | 368 (7.3) | 1.17 (1.06-1.29)† | 1.06 (0.96-1.17) | 656 (13.1) | 1.34 (1.25-1.44)‡ | 1.18 (1.10-1.27) ‡ |
| Circulatory system |  |  |  |  |  |  |
| Absent (N = 855,508) | 53,787 (6.3) | 1.00 (referent) | 1.00 (referent) | 83,250 (9.7) | 1.00 (referent) | 1.00 (referent) |
| Present (N = 2,496) | 177 (7.1) | 1.13 (0.98-1.30) | 1.06 (0.92-1.22) | 314 (12.6) | 1.29 (1.16-1.43) ‡ | 1.20 (1.08-1.33) ‡ |
| Respiratory system |  |  |  |  |  |  |
| Absent (N = 845,562) | 52,969 (6.3) | 1.00 (referent) | 1.00 (referent) | 81,764 (9.7) | 1.00 (referent) | 1.00 (referent) |
| Present (N = 12,442) | 995 (8.0) | 1.27 (1.20-1.35) ‡ | 1.15 (1.08-1.22) ‡ | 1,800 (14.5) | 1.49 (1.42-1.55) ‡ | 1.28 (1.22-1.34) ‡ |
| Musculoskeletal system |  |  |  |  |  |  |
| Absent (N = 854,010) | 53,663 (6.3) | 1.00 (referent) | 1.00 (referent) | 83,057 (9.7) | 1.00 (referent) | 1.00 (referent) |
| Present (N = 3,994) | 301 (7.5) | 1.20 (1.07-1.33) † | 1.12 (1.00-1.25) * | 507 (12.7) | 1.29 (1.19-1.40) ‡ | 1.19 (1.10-1.29) ‡ |
| Nervous system and sense organs |  |  |  |  |  |  |
| Absent (N = 845,978) | 52,970 (6.3) | 1.00 (referent) | 1.00 (referent) | 81,894 (9.7) | 1.00 (referent) | 1.00 (referent) |
| Present (N = 12,026) | 994 (8.3) | 1.31 (1.24-1.40) ‡ | 1.22 (1.15-1.30) ‡ | 1,670 (13.9) | 1.43 (1.36-1.49) ‡ | 1.25 (1.20-1.31) ‡ |
| Digestive system |  |  |  |  |  |  |
| Absent (N = 847,652) | 53,152 (6.3) | 1.00 (referent) | 1.00 (referent) | 82,145 (9.7) | 1.00 (referent) | 1.00 (referent) |
| Present (N = 10,352) | 812 (7.8) | 1.25 (1.17-1.33) ‡ | 1.16 (1.08-1.23) ‡ | 1,419 (13.7) | 1.41 (1.34-1.48) ‡ | 1.27 (1.21-1.33) ‡ |
| Genitourinary system |  |  |  |  |  |  |
| Absent (N = 823,562) | 51,496 (6.3) | 1.00 (referent) | 1.00 (referent) | 79,228 (9.6) | 1.00 (referent) | 1.00 (referent) |
| Present (N = 34,442) | 2,468 (7.2) | 1.14 (1.10-119) ‡ | 1.06 (1.02-1.10) † | 4,336 (12.6) | 1.30 (1.26-1.34) ‡ | 1.18 (1.15-1.22) ‡ |
| Diseases of the skin and subcutaneous tissue |  |  |  |  |  |  |
| Absent (N = 857,568) | 53,937 (6.3) | 1.00 (referent) | 1.00 (referent) | 83,511 (9.7) | 1.00 (referent) | 1.00 (referent) |
| Present (N = 436) | 27 (6.2) | 0.98 (0.68-1.41) | 0.88 (0.61-1.27) | 53 (12.2) | 1.24 (0.96-1.59) | 1.04 (0.81-1.34) |
| Diseases of the blood and blood-forming organs |  |  |  |  |  |  |
| Absent (N = 857,232) | 53,902 (6.3) | 1.00 (referent) | 1.00 (referent) | 83,470 (9.7) | 1.00 (referent) | 1.00 (referent) |
| Present (N = 772) | 62 (8.0) | 1.27 (0.99-1.61) | 1.14 (0.89-1.45) | 94 (12.2) | 1.25 (1.03-1.51) * | 1.11 (0.92-1.33) |
| Neoplasms |  |  |  |  |  |  |
| Absent (N = 856,957) | 53,887 (6.3) | 1.00 (referent) | 1.00 (referent) | 83,459 (9.7) | 1.00 (referent) | 1.00 (referent) |
| Present (N = 1,047) | 77 (7.4) | 1.16 (0.94-1.44) | 1.11 (0.90-1.38) | 105 (10.0) | 1.03 (0.86-1.23) | 0.98 (0.82-1.18) |
| Infections |  |  |  |  |  |  |
| Absent (N = 857,386) | 53,908 (6.3) | 1.00 (referent) | 1.00 (referent) | 83,474 (9.7) | 1.00 (referent) | 1.00 (referent) |
| Present (N = 618) | 56 (9.1) | 1.43 (1.12-1.84) † | 1.23 (0.96-1.58) | 90 (14.6) | 1.49 (1.23-1.80) ‡ | 1.15 (0.95-1.40) |
| Congenital anomalies |  |  |  |  |  |  |
| Absent (N = 855,628) | 53,792 (6.3) | 1.00 (referent) | 1.00 (referent) | 83,309 (9.7) | 1.00 (referent) | 1.00 (referent) |
| Present (N = 2,376) | 172 (7.2) | 1.15 (0.99-1.33) | 1.05 (0.91-1.21) | 255 (10.7) | 1.10 (0.98-1.23) | 0.98 (0.87-1.10) |
| Injury and poisoning |  |  |  |  |  |  |
| Absent (N = 857,844) | 53,955 (6.3) | 1.00 (referent) | 1.00 (referent) | 83,550 (9.7) | 1.00 (referent) | 1.00 (referent) |
| Present (N = 160) | 9 (5.6) | 0.90 (0.48-1.68) | 0.77 (0.39-1.49) | 14 (8.8) | 0.91 (0.55-1.48) | 0.82 (0.50-1.35) |

^a^ Adjusted for age, parity, rural residence, neighbourhood income quintile, remote history of mental health care, and the presence of other chronic physical conditions.

* = p<.05, † = p<.01, ‡ = p<.001
