# Supplementary material for: Chronic physical conditions and risk for perinatal mental illness: A population-based retrospective cohort study
Source: PLoS Med. 2019 Aug 26;16(8):e1002864. doi: 10.1371/journal.pmed.1002864 (PMC6709891; doi:10.1371/journal.pmed.1002864)
Supplement: S6 Table — (DOCX) [file pmed.1002864.s008.docx]

**S6 Table.** **Risk of a psychotic disorder or mood or anxiety disorder arising between conception and 1 year postpartum, in relation to a woman having a chronic physical condition in the 24 months prior to conception, and further detailed by the type of chronic physical condition.**

|  | **Psychotic disorder** | | | **Mood or anxiety disorder** | | |
| --- | --- | --- | --- | --- | --- | --- |
| **Variable** | **Number (%) with outcome** | **Unadjusted relative risk (95% CI)** | **Adjusted relative risk (95% CI)^a^** | **Number (%) with outcome** | **Unadjusted relative risk (95% CI)** | **Adjusted relative risk (95% CI)^a^** |
| Endocrine and metabolic disorders |  |  |  |  |  |  |
| Absent (N = 852,990) | 3,197 (0.37) | 1.00 (referent) | 1.00 (referent) | 132,249 (15.5) | 1.00 (referent) | 1.00 (referent) |
| Present (N = 5,014) | 33 (0.66) | 1.74 (1.24-2.45) ‡ | 1.42 (1.01-2.01)* | 971 (19.4) | 1.24 (1.17-1.31) ‡ | 1.12 (1.06-1.19) ‡ |
| Circulatory system |  |  |  |  |  |  |
| Absent (N = 855,508) | 3,214 (0.38) | 1.00 (referent) | 1.00 (referent) | 132,751 (15.5) | 1.00 (referent) | 1.00 (referent) |
| Present (N = 2,496) | 16 (0.64) | 1.71 (1.05-2.77) | 1.50 (0.92-2.44) | 469 (18.8) | 1.21 (1.11-1.31) ‡ | 1.13 (1.04-1.22) ‡ |
| Respiratory system |  |  |  |  |  |  |
| Absent (N = 845,562) | 3,133 (0.37) | 1.00 (referent) | 1.00 (referent) | 130,551 (15.4) | 1.00 (referent) | 1.00 (referent) |
| Present (N = 12,442) | 97 (0.78) | 2.09 (1.71-2.56) ‡ | 1.69 (1.38-2.07) ‡ | 2,669 (21.5) | 1.37 (1.32-1.42) ‡ | 1.22 (1.18-1.26) ‡ |
| Musculoskeletal system |  |  |  |  |  |  |
| Absent (N = 854,010) | 3,217 (0.38) | 1.00 (referent) | 1.00 (referent) | 132,442 (15.5) | 1.00 (referent) | 1.00 (referent) |
| Present (N = 3,994) | 13 (0.33) | 0.85 (0.49-1.47) | 0.76 (0.44-1.31) | 778 (19.5) | 1.24 (1.16-1.32) ‡ | 1.15 (1.08-1.23) ‡ |
| Nervous system and sense organs |  |  |  |  |  |  |
| Absent (N = 845,978) | 3,151 (0.37) | 1.00 (referent) | 1.00 (referent) | 130,683 (15.5) | 1.00 (referent) | 1.00 (referent) |
| Present (N = 12,026) | 79 (0.66) | 1.75 (1.40-2.19) ‡ | 1.45 (1.15-1.82) ‡ | 2,537 (21.1) | 1.35 (1.30-1.39) ‡ | 1.23 (1.19-1.27) ‡ |
| Digestive system |  |  |  |  |  |  |
| Absent (N = 847,652) | 3,166 (0.37) | 1.00 (referent) | 1.00 (referent) | 131,075 (15.5) | 1.00 (referent) | 1.00 (referent) |
| Present (N = 10,352) | 64 (0.62) | 1.64 (1.29-2.10) ‡ | 1.41 (1.10-1.81) ‡ | 2,145 (20.7) | 1.32 (1.27-1.37) ‡ | 1.22 (1.17-1.26) ‡ |
| Genitourinary system |  |  |  |  |  |  |
| Absent (N = 823,562) | 3,027 (0.37) | 1.00 (referent) | 1.00 (referent) | 126,682 (15.4) | 1.00 (referent) | 1.00 (referent) |
| Present (N = 34,442) | 203 (0.59) | 1.59 (1.38-1.83) ‡ | 1.37 (1.19-1.58) ‡ | 6,538 (19.0) | 1.22 (1.19-1.24) ‡ | 1.12 (1.10-1.15) ‡ |
| Diseases of the skin and subcutaneous tissue |  |  |  |  |  |  |
| Absent (N = 857,568) | --- | 1.00 (referent) | 1.00 (referent) | 133,144 (15.5) | 1.00 (referent) | 1.00 (referent) |
| Present (N = 436) | --- | --- | --- | 76 (17.4) | 1.10 (0.90-1.35) | 0.97 (0.79-1.19) |
| Diseases of the blood and blood-forming organs |  |  |  |  |  |  |
| Absent (N = 857,232) | --- | 1.00 (referent) | 1.00 (referent) | 133,068 (15.5) | 1.00 (referent) | 1.00 (referent) |
| Present (N = 772) | --- | --- | --- | 152 (19.7) | 1.26 (1.09-1.45) ‡ | 1.13 (0.98-1.30) |
| Neoplasms |  |  |  |  |  |  |
| Absent (N = 856,957) | --- | 1.00 (referent) | 1.00 (referent) | 133,042 (15.5) | 1.00 (referent) | 1.00 (referent) |
| Present (N = 1,047) | --- | --- | --- | 178 (17.0) | 1.08 (0.95-1.23) | 1.04 (0.91-1.19) |
| Infections |  |  |  |  |  |  |
| Absent (N = 857,386) | --- | 1.00 (referent) | 1.00 (referent) | 133,089 (15.5) | 1.00 (referent) | 1.00 (referent) |
| Present (N = 618) | --- | --- | --- | 131 (21.2) | 1.35 (1.16-1.57) ‡ | 1.11 (0.95-1.29) |
| Congenital anomalies |  |  |  |  |  |  |
| Absent (N = 855,628) | 3,223 (0.38) | 1.00 (referent) | 1.00 (referent) | 132,807 (15.5) | 1.00 (referent) | 1.00 (referent) |
| Present (N = 2,376) | 7 (0.29) | 0.79 (0.38-1.64) | 0.66 (0.32-1.36) | 413 (17.4) | 1.11 (1.02-1.21) * | 1.00 (0.92-1.09) |
| Injury and poisoning |  |  |  |  |  |  |
| Absent (N = 857,844) | --- | 1.00 (referent) | 1.00 (referent) | 133,199 (15.5) | 1.00 (referent) | 1.00 (referent) |
| Present (N = 160) | --- | --- | --- | 21 (13.1) | 0.86 (0.59-1.26) | 0.77 (0.52-1.14) |

-- = data suppressed to protect patient privacy, due to cell sizes < 6.

^a^ Adjusted for age, parity, rural residence, neighbourhood income quintile, remote history of mental health care, and the presence of other chronic physical conditions.

* = p<.05, † = p<.01, ‡ = p<.001
