# Supplementary material for: Chronic physical conditions and risk for perinatal mental illness: A population-based retrospective cohort study
Source: PLoS Med. 2019 Aug 26;16(8):e1002864. doi: 10.1371/journal.pmed.1002864 (PMC6709891; doi:10.1371/journal.pmed.1002864)
Supplement: S7 Table — (DOCX) [file pmed.1002864.s009.docx]

**S7 Table.** **Risk of a substance use disorder, self-harm, and other mental illness arising between conception and 1 year postpartum, in relation to a woman having a chronic physical condition in the 24 months prior to conception, and further detailed by the type of chronic physical condition.**

|  | **Substance use disorder** | | | **Self-harm** | | | **Other** | | |
| --- | --- | --- | --- | --- | --- | --- | --- | --- | --- |
| **Variable** | **Number (%) with outcome** | **Unadjusted relative risk (95% CI)** | **Adjusted relative risk (95% CI)^a^** | **Number (%) with outcome** | **Unadjusted relative risk (95% CI)** | **Adjusted relative risk (95% CI)^a^** | **Number (%) with outcome** | **Unadjusted relative risk (95% CI)** | **Adjusted relative risk (95% CI)^a^** |
| Endocrine and metabolic disorders |  |  |  |  |  |  |  |  |  |
| Absent (N = 852,990) | 3,266 (0.38) | 1.00 (referent) | 1.00 (referent) | 540 (0.06) | 1.00 (referent) | 1.00 (referent) | 2,440 (0.29) | 1.00 (referent) | 1.00 (referent) |
| Present (N = 5,014) | 47 (0.94) | 2.43 (1.82-3.25) ‡ | 1.68 (1.24-2.29) ‡ | 8 (0.16) | 2.52 (1.26-5.04) † | 2.09 (0.98-4.44) | 23 (0.46) | 1.58 (1.05-2.38) * | 1.25 (0.83-1.89) |
| Circulatory system |  |  |  |  |  |  |  |  |  |
| Absent (N = 855,508) | 3,298 (0.39) | 1.00 (referent) | 1.00 (referent) |  | 1.00 (referent) | 1.00 (referent) | 2,452 (0.29) | 1.00 (referent) | 1.00 (referent) |
| Present (N = 2,496) | 15 (0.60) | 1.48 (0.88-2.49) | 1.27 (0.75-2.16) | --- | --- | --- | 11 (0.44) | 1.52 (0.85-2.73) | 1.32 (0.74-2.38) |
| Respiratory system |  |  |  |  |  |  |  |  |  |
| Absent (N = 845,562) | 3,193 (0.38) | 1.00 (referent) | 1.00 (referent) | 539 (0.06) | 1.00 (referent) | 1.00 (referent) | 2,396 (0.28) | 1.00 (referent) | 1.00 (referent) |
| Present (N = 12,442) | 120 (0.96) | 2.48 (2.07-2.99) ‡ | 1.41 (1.17-1.71) ‡ | 9 (0.07) | 1.08 (0.52-2.23) | 0.70 (0.34-1.45) | 67 (0.54) | 1.87 (1.46-2.39) ‡ | 1.38 (1.08-1.77) * |
| Musculoskeletal system |  |  |  |  |  |  |  |  |  |
| Absent (N = 854,010) | 3,283 (0.38) | 1.00 (referent) | 1.00 (referent) |  | 1.00 (referent) | 1.00 (referent) | 2,444 (0.29) | 1.00 (referent) | 1.00 (referent) |
| Present (N = 3,994) | 30 (0.75) | 1.93 (1.35-2.76) ‡ | 1.62 (1.12-2.32) † | --- | --- | --- | 19 (0.48) | 1.66 (1.07-2.60) * | 1.38 (0.87-2.18) |
| Nervous system and sense organs |  |  |  |  |  |  |  |  |  |
| Absent (N = 845,978) | 3,202 (0.38) | 1.00 (referent) | 1.00 (referent) | 530 (0.06) | 1.00 (referent) | 1.00 (referent) | 2,386 (0.28) | 1.00 (referent) | 1.00 (referent) |
| Present (N = 12,026) | 111 (0.92) | 2.37 (1.96-2.87) ‡ | 1.43 (1.17-1.74) ‡ | 18 (0.15) | 2.40 (1.51-3.82) ‡ | 1.71 (1.07-3.75) | 77 (0.64) | 2.24 (1.78-2.81) ‡ | 1.75 (1.38-2.20) ‡ |
| Digestive system |  |  |  |  |  |  |  |  |  |
| Absent (N = 847,652) | 3,249 (0.38) | 1.00 (referent) | 1.00 (referent) | 540 (0.06) | 1.00 (referent) | 1.00 (referent) | 2,401 (0.28) | 1.00 (referent) | 1.00 (referent) |
| Present (N = 10,352) | 64 (0.62) | 1.57 (1.22-2.01) ‡ | 1.17 (0.91-1.51) | 8 (0.08) | 1.19 (0.59-2.42) | 1.01 (0.50-2.04) | 62 (0.60) | 2.09 (1.62-2.71) ‡ | 1.76 (1.36-2.28) ‡ |
| Genitourinary system |  |  |  |  |  |  |  |  |  |
| Absent (N = 823,562) | 3,088 (0.37) | 1.00 (referent) | 1.00 (referent) | 520 (0.06) | 1.00 (referent) | 1.00 (referent) | 2,283 (0.28) | 1.00 (referent) | 1.00 (referent) |
| Present (N = 34,442) | 225 (0.65) | 1.70 (1.48-1.94) ‡ | 1.27 (1.11-1.46) ‡ | 28 (0.08) | 1.27 (0.87-1.86) | 0.99 (0.67-1.45) | 180 (0.52) | 1.86 (1.60-2.17) ‡ | 1.55 (1.33-1.80) ‡ |
| Diseases of the skin and subcutaneous tissue |  |  |  |  |  |  |  |  |  |
| Absent (N = 857,568) | --- | 1.00 (referent) | 1.00 (referent) | 548 (0.06) | 1.00 (referent) | 1.00 (referent) | 2,457 (0.29) | 1.00 (referent) | 1.00 (referent) |
| Present (N = 436) | --- | --- | --- | 0 (0.0) | N/A | N/A | 6 (1.4) | 4.81 (2.19-10.55) ‡ | 3.47 (1.58-7.73) ‡ |
| Diseases of the blood and blood-forming organs |  |  |  |  |  |  |  |  |  |
| Absent (N = 857,232) | 3,307 (0.39) | 1.00 (referent) | 1.00 (referent) | 548 (0.06) | 1.00 (referent) | 1.00 (referent) | --- | 1.00 (referent) | 1.00 (referent) |
| Present (N = 772) | 6 (0.78) | 2.02 (0.92-4.44) | 1.48 (0.68-3.25) | 0 (0.0) | N/A | N/A | --- | --- | --- |
| Neoplasms |  |  |  |  |  |  |  |  |  |
| Absent (N = 856,957) | --- | 1.00 (referent) | 1.00 (referent) | 548 (0.06) | 1.00 (referent) | 1.00 (referent) | --- | 1.00 (referent) | 1.00 (referent) |
| Present (N = 1,047) | --- | --- | --- | 0 (0.0) | N/A | N/A | --- | --- | --- |
| Infections |  |  |  |  |  |  |  |  |  |
| Absent (N = 857,386) | 3,297 (0.38) | 1.00 (referent) | 1.00 (referent) | --- | 1.00 (referent) | 1.00 (referent) | 2,455 (0.29) | 1.00 (referent) | 1.00 (referent) |
| Present (N = 618) | 16 (2.59) | 6.48 (3.96-10.62) ‡ | 2.93 (1.80-4.75) ‡ | --- | --- | --- | 8 (1.3) | 4.50 (2.28-8.89) ‡ | 2.81 (1.41-5.59) ‡ |
| Congenital anomalies |  |  |  |  |  |  |  |  |  |
| Absent (N = 855,628) | 3,304 (0.39) | 1.00 (referent) | 1.00 (referent) |  | 1.00 (referent) | 1.00 (referent) | 2,455 (0.29) | 1.00 (referent) | 1.00 (referent) |
| Present (N = 2,376) | 9 (0.38) | 0.97 (0.51-1.86) | 1.10 (0.58-2.10) | --- | --- | --- | 8 (0.34) | 1.16 (0.58-2.32) | 0.96 (0.48-1.93) |
| Injury and poisoning |  |  |  |  |  |  |  |  |  |
| Absent (N = 857,844) | --- | 1.00 (referent) | 1.00 (referent) | --- | 1.00 (referent) | 1.00 (referent) | 2,463 (0.29) | 1.00 (referent) | 1.00 (referent) |
| Present (N = 160) | --- | --- | --- | --- | --- | --- | 0 (0.0) | N/A | N/A |

-- = data suppressed to protect patient privacy, due to cell sizes < 6.

^a^ Adjusted for age, parity, rural residence, neighbourhood income quintile, remote history of mental health care, and the presence of other chronic physical conditions.

* = p<.05, † = p<.01, ‡ = p<.001
