# Supplementary material for: Chronic physical conditions and risk for perinatal mental illness: A population-based retrospective cohort study
Source: PLoS Med. 2019 Aug 26;16(8):e1002864. doi: 10.1371/journal.pmed.1002864 (PMC6709891; doi:10.1371/journal.pmed.1002864)
Supplement: S8 Table — (DOCX) [file pmed.1002864.s010.docx]

**S8 Table. Risk of perinatal mental illness requiring at least two physician visits between conception and 1 year postpartum, in relation to a woman having a chronic physical condition in the 24 months prior to conception.**

| **Variable** | **Number (%) with outcome** | **Unadjusted** | | **Adjusted^a^** | |
| --- | --- | --- | --- | --- | --- |
|  |  | **Relative risk (95% CI)** | **p-value** | **Relative risk (95% CI)** | **p-value** |
| **Main exposure of interest** |  |  |  |  |  |
| No chronic physical condition (N = 780,619) | 50,999 (6.5) | 1.00 (referent) |  | 1.00 (referent) |  |
| Chronic physical condition (N = 77,385) | 7,499 (9.7) | 1.47 (1.43-1.50) | <.0001 | 1.31 (1.28-1.34) | <.0001 |
| Age, years |  |  |  |  |  |
| 15-24 | 14,496 (9.8) | 1.56 (1.53-1.59) | <.0001 | 1.55 (1.52-1.58) | <.0001 |
| 25-34 | 34,513 (6.2) | 1.00 (referent) |  | 1.00 (referent) |  |
| 35-49 | 9,489 (6.1) | 0.98 (0.96-1.00) | .11 | 0.96 (0.94-0.99) | .0012 |
| Parity |  |  |  |  |  |
| Primiparous | 29,105 (7.7) | 1.00 (referent) |  | 1.00 (referent) |  |
| Multiparous | 29,392 (6.1) | 0.82 (0.80-0.83) | <.0001 | 0.83 (0.82-0.84) | <.0001 |
| Region of residence |  |  |  |  |  |
| Urban | 51,829 (6.8) | 1.00 (referent) |  | 1.00 (referent) |  |
| Rural | 6,662 (7.1) | 1.05 (1.02-1.07) | .0003 | 0.97 (0.95-1.00) | .04 |
| Neighbourhood income quintile (Q) |  |  |  |  |  |
| Q1 (lowest) | 13,562 (7.6) | 1.20 (1.16-1.23) | <.0001 | 1.13 (1.10-1.16) | <.0001 |
| Q2 | 11,884 (6.9) | 1.09 (1.06-1.12) | <.0001 | 1.06 (1.03-1.09) | <.0001 |
| Q3 | 11,753 (6.7) | 105 (1.03-1.08) | .0001 | 1.04 (1.01-1.06) | .01 |
| Q4 | 11,925 (6.5) | 1.03 (1.00-1.06) | .03 | 1.03 (1.00-1.05) | .06 |
| Q5 (highest) | 9,022 (6.3) | 1.00 (referent) |  | 1.00 (referent) |  |
| Remote history of mental illness more than 2 years preceding the index birth |  |  |  |  |  |
| Absent | 19,997 (4.5) | 1.00 (referent) |  | 1.00 (referent) |  |
| Present | 38,501 (9.4) | 2.07 (2.04-2.11) | <.0001 | 2.16 (2.12-2.19) | <.0001 |

^a^ This narrower definition of perinatal mental illness required at least two physician visits, or one or more emergency department visits or hospitalizations, for a mental health reason within pregnancy or 365 days postpartum. This ensured that the first physician visit could not be for assessment rather than diagnosis.

^b^ Adjusted for age, parity, rural residence, neighbourhood income quintile, and remote history of mental health care.
